# Regulation of microglial responses after pediatric traumatic brain injury: exploring the role of SHIP-1

**DOI:** 10.3389/fnins.2023.1276495

**Published:** 2023-10-13

**Authors:** Erskine Chu, Richelle Mychasiuk, Tabitha R. F. Green, Akram Zamani, Larissa K. Dill, Rishabh Sharma, April L. Raftery, Evelyn Tsantikos, Margaret L. Hibbs, Bridgette D. Semple

**Affiliations:** ^1^Department of Neuroscience, Monash University, Melbourne, VIC, Australia; ^2^Department of Immunology, Monash University, Melbourne, VIC, Australia; ^3^Deparment of Neurology, Alfred Health, Prahran, VIC, Australia; ^4^Department of Integrative Physiology, The University of Colorado Boulder, Boulder, CO, United States; ^5^Alfred Health, Prahran, VIC, Australia; ^6^Department of Medicine (Royal Melbourne Hospital), The University of Melbourne, Parkville, VIC, Australia

**Keywords:** immune responses, inflammation, neurotrauma, immune signaling, PI3K

## Abstract

**Introduction:**

Severe traumatic brain injury (TBI) is the world’s leading cause of permanent neurological disability in children. TBI-induced neurological deficits may be driven by neuroinflammation post-injury. Abnormal activity of SH2 domain-containing inositol 5′ phosphatase-1 (SHIP-1) has been associated with dysregulated immunological responses, but the role of SHIP-1 in the brain remains unclear. The current study investigated the immunoregulatory role of SHIP-1 in a mouse model of moderate–severe pediatric TBI.

**Methods:**

SHIP-1+/− and SHIP-1−/− mice underwent experimental TBI or sham surgery at post-natal day 21. Brain gene expression was examined across a time course, and immunofluorescence staining was evaluated to determine cellular immune responses, alongside peripheral serum cytokine levels by immunoassays. Brain tissue volume loss was measured using volumetric analysis, and behavior changes both acutely and chronically post-injury.

**Results:**

Acutely, inflammatory gene expression was elevated in the injured cortex alongside increased IBA-1 expression and altered microglial morphology; but to a similar extent in SHIP-1−/− mice and littermate SHIP-1+/− control mice. Similarly, the infiltration and activation of CD68-positive macrophages, and reactivity of GFAP-positive astrocytes, was increased after TBI but comparable between genotypes. TBI increased anxiety-like behavior acutely, whereas SHIP-1 deficiency alone reduced general locomotor activity. Chronically, at 12-weeks post-TBI, SHIP-1−/− mice exhibited reduced body weight and increased circulating cytokines. Pro-inflammatory gene expression in the injured hippocampus was also elevated in SHIP-1−/− mice; however, GFAP immunoreactivity at the injury site in TBI mice was lower. TBI induced a comparable loss of cortical and hippocampal tissue in both genotypes, while SHIP-1−/− mice showed reduced general activity and impaired working memory, independent of TBI.

**Conclusion:**

Together, evidence does not support SHIP-1 as an essential regulator of brain microglial morphology, brain immune responses, or the extent of tissue damage after moderate–severe pediatric TBI in mice. However, our data suggest that reduced SHIP-1 activity induces a greater inflammatory response in the hippocampus chronically post-TBI, warranting further investigation.

## Introduction

Despite the fact that the developing brain has a high capacity for plasticity, traumatic brain injury (TBI) is one of the largest global pediatric health concerns as the leading cause of permanent neurological disability and death in children and adolescents (Dewan et al., 2016). Survivors of pediatric TBI often exhibit persistent symptoms such as depression, anxiety, learning and memory deficits, and social problems, both during the acute post-injury period as well as into adulthood (Babikian and Asarnow, 2009; Max, 2014; Stephens et al., 2017). The limited recovery after a moderate or severe TBI, in both clinical studies and animal models, is likely a consequence of additional neuropathology beyond the initial injury site, which may evolve over time and be influenced by age at the time of injury (Kolb et al., 2000; Anderson et al., 2011).

The lipid phosphatase SHIP-1 is a key regulator of lymphocyte and myeloid cell activation in the periphery, and its loss of function is associated with several inflammatory diseases (Hibbs et al., 2018). SHIP-1 is highly expressed in microglia (Zhang et al., 2014, 2016; Marsh et al., 2022), yet it’s potential regulatory role in neuroinflammation in the context of injury or disease states remains unclear. Microglia are a distinct component of the innate immune system that function as brain-resident mononuclear phagocytes, and are highly comparable to macrophages in their function and biomarker expression (Donat et al., 2017). In response to brain injury, microglia undergo morphological changes and demonstrate both pro- and anti-inflammatory responses (Hickman et al., 2013). There is increasing evidence to suggest that aberrant microglial responses, both proximal and distal to the injury site, promote prolonged neuroinflammation that contributes to secondary neuropathology after TBI (Raghavendra Rao et al., 2000; Tasker et al., 2005; Wilde et al., 2007; Mahajan et al., 2011; Kostandy, 2012; Johnson et al., 2013; Loane et al., 2014; Viviani et al., 2014; Girgis et al., 2016; Kinoshita, 2016; Raghupathi and Huh, 2017). Additionally, depletion of microglia after TBI in adult rodent models has been shown to reduce neuropathology and improve cognitive outcomes (Rice et al., 2015; Henry et al., 2020; Cai et al., 2022). However, the underlying regulators of microglial reactivity and plasticity are unclear.

The phosphatidylinositol-3-kinase (PI3K)-serine/threonine-specific protein kinase (AKT) signaling cascade mediates survival, proliferation, differentiation, migration, and metabolism in various cell types, including leukocytes (Zhou et al., 2000; Scheid and Woodgett, 2001; Chang et al., 2003; Koyasu, 2003; Xiao et al., 2010; Xie et al., 2014). In hematopoietic cells, this pathway is, in part, negatively regulated by SHIP-1 through dephosphorylation of the lipid-derived second messenger phosphatidylinositol 3,4,5-trisphosphate (Hibbs et al., 2018; Chu et al., 2021). Loss of SHIP-1 activity drives extramedullary hematopoiesis and has been implicated in pronounced chronic inflammatory diseases, such as inflammatory lung disease, osteoporosis, lupus, and Crohn’s-like ileitis (Helgason et al., 1998; Liu et al., 1999; Takeshita et al., 2002; Kerr et al., 2011; Maxwell et al., 2011; McLarren et al., 2011; Hibbs et al., 2018). These findings highlight the critical role of SHIP-1 as a negative regulator of immune cell signaling.

Emerging evidence indicates that SHIP-1 helps maintain homeostasis in the central nervous system. Microglia and brain endothelial cells from humans and mice have been shown to express considerable levels of SHIP-1 (Olah et al., 2018; Pedicone et al., 2020). Single nucleotide polymorphisms within the human SHIP-1 gene *INPP5D* are strongly correlated with the development of Alzheimer’s disease and associated pathology in the aging brain (Farfel et al., 2016; Jing et al., 2016; Efthymiou and Goate, 2017; Yoshino et al., 2017; Tsai et al., 2021; Zajac et al., 2021). In addition, heightened expression of *Inpp5d* in plaque-associated microglia from preclinical Alzheimer’s Disease models implicate the SHIP-1 pathway in plaque clearance (Lin et al., 2021; Tsai et al., 2021). These findings suggest that SHIP-1 may have a neuroprotective role in the brain by regulating microglial responses.

Given the role of neuroinflammation in the modulation of pediatric TBI outcomes, and the significant function of SHIP-1 in the regulation of peripheral inflammation, we hypothesized that SHIP-1 would negatively regulate microglial responses following pediatric TBI. To address this hypothesis, SHIP-1-deficient mice underwent experimental TBI or sham surgery, and we examined neuroimmunological, pathological, and behavioral outcomes across an extended time course post-injury.

## Methods and methods

### Animals and ethics

Male and female SHIP-1-deficient mice (*Inpp5d^tm1Dmt^*) (Liu et al., 1999) mice on a C57BL/6 background were used (Maxwell et al., 2011). For all studies, littermate SHIP-1+/− mice were used as controls, as they have previously been extensively characterized as exhibiting an identical peripheral inflammatory phenotype to SHIP-1+/+ mice (Maxwell et al., 2011). In addition, analysis of acute TBI responses in SHIP-1+/+ and SHIP-1+/− indicated comparable immunological responses to brain injury (Supplementary Figure S1 and Supplementary Table S1). Mice were generated by breeding heterozygous SHIP-1+/− females with homozygous SHIP-1−/− males, and genotypes were confirmed by PCR, with the distribution of experimental animals shown in Supplementary Table S2. Mice were housed in a specific pathogen-free facility in the Precinct Animal Center at the Alfred Research Alliance under a 12-h light–dark cycle (lights on at 0700), with unrestricted access to food and water. All surgical procedures were approved by the Alfred Research Alliance Animal Ethics Committee (#E-1881-2019-M and #E-8259-2022-M) and were conducted in accordance with the guidelines of the Australian Code of Practice for the Care and Use of Animals for Scientific Purposes.

### Experimental design

**Figure 1:**
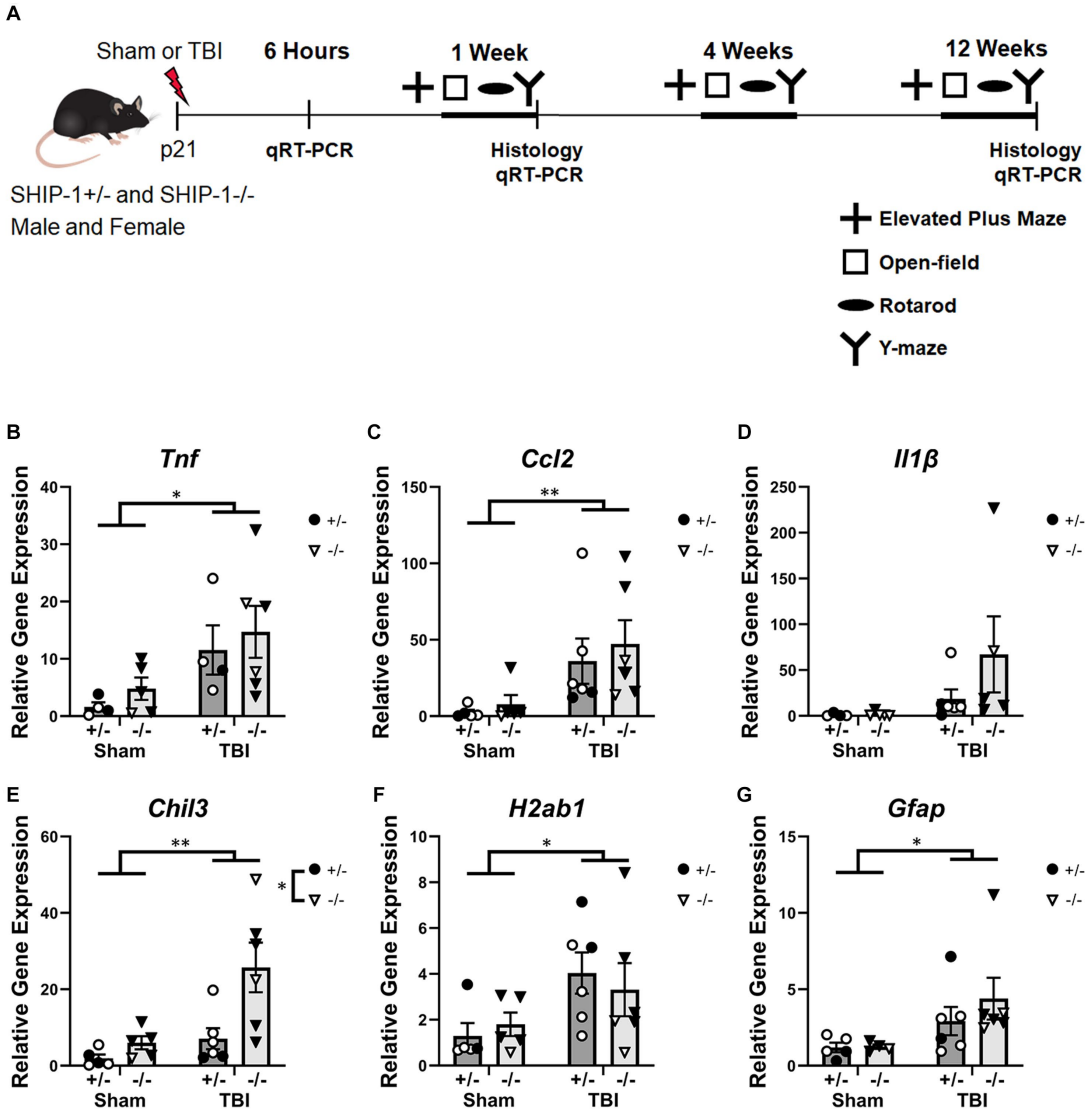
TBI increased expression of inflammatory genes at 6 h post-injury. **(A)** Experimental timeline. **(B)** Relative expression of the indicated genes in the ipsilateral cortex from SHIP-1+/− and SHIP-1−/− mice, at 6 h post-TBI/sham surgeries **(B–G)**. *p < 0.05 **p < 0.01, two-way ANOVA main effect of injury; *n* = 4-6/group. Complete reporting of statistical analyzes is in Supplementary Table S4.

SHIP-1+/− and SHIP-1−/− littermate mice underwent sham or experimental TBI surgeries using the CCI model at postnatal day 21 (p21) ± 1 day. Neurobehavioral outcomes were then assessed at 1-week, 4-weeks, and 12-weeks after surgery. Tissue was collected at 6 h post-injury for gene expression analysis, and at 1-week and 12-weeks post-injury for histology and gene expression analysis (Figure 1A). Mice were randomized to receive either TBI or sham surgery, and all outcome measures and analyzes were conducted by investigators blinded to genotype and injury group.

### Controlled cortical impact

Controlled Cortical Impact (CCI) was performed as previously described (Tong et al., 2002; Semple et al., 2017). Briefly, mice were anesthetized with 4% isoflurane in O_2_ gas *via* a nose cone, with anesthesia maintained at 1.5–2% isoflurane for the duration of the procedure. The head was fixed in a stereotaxic frame, bupivacaine (1 mg/kg s.c.) was administered locally to the mid-line of the scalp, and buprenorphine (0.5 mg/kg s.c.) was injected to the animal’s flank. A unilateral 3.5 mm craniectomy was performed using a fine-tipped dental drill (Ideal Micro-drill, Cellpoint Scientific, Gaithersburg, United States). A moderate-to-severe CCI was induced with an electric Cortical Contusion Impactor (Custom Design & Fabrication, Sandston, United States) at an impactor velocity of 4.5 m/s and a deformation depth of 1.7 mm with a 3 mm diameter tip, to the left parietal lobe. Sham mice underwent an identical surgical procedure to TBI mice (i.e., anesthesia, surgical preparation, and craniectomy), but did not receive the TBI impact. Post-operatively, mice were administered subcutaneous sterile isotonic saline to assist with rehydration and maintained on heated pads until self-righting, before being returned to group-housing with same-sex littermates (mix of both TBI and sham). Two SHIP-1−/− animals were excluded as they failed to recover following surgery.

### Behavior testing

Behavior testing was conducted at 1-week, 4-weeks, and 12-weeks post-surgery. Anxiety-like behavior was assessed using the Elevated Plus Maze (10 min of testing). General locomotor activity and anxiety levels were analyzed using an open-field test (10 min of testing). Gross motor functioning and coordination were assessed using the accelerating rotarod test over two subsequent days. Working and spatial memory were evaluated using the Y-maze (15 min of habituating, 30 min inter-trial interval, 5 min of testing) and the discrimination index $\left(\mathrm{Discrimination}\;\mathrm{index}=\frac{Time_{\mathrm{Novel}}-Time_{\mathrm{Familiar}}}{Time_{\mathrm{Novel}}+Time_{\mathrm{Familiar}}}\right)$ . All tests were performed as detailed previously (Sharma et al., 2022), by an investigator blinded to the experimental group.

### Transcardial perfusion and tissue fixation

Transcardial perfusion and tissue fixation procedures were performed as previously described (Gage et al., 2012). Briefly, mice were euthanized with sodium pentobarbitone (80 mg/kg i.p.), spleens were extracted and weighed, and blood was collected from the right atrium for serum isolation. Mice then underwent transcardial perfusion with saline (3 mL/min for 5 min) followed by 4% paraformaldehyde. The collected brain was post-fixed in 4% paraformaldehyde overnight. Brains collected at 1-week post-injury were immersed in 30% sucrose for 3–5 days before being embedded in Optimal Cutting Temperature (OCT) media (ProSciTech, Kirwan, Australia) for sectioning. Brains collected at 12-weeks post-injury were immersed in 70% ethanol for 2 days then embedded in paraffin for sectioning.

### Immunofluorescence

Perfused brains were sectioned from approximately Bregma 0.7 mm to 3.5 mm. 12 μm thick coronal sections were cut from OCT-embedded brains and 8 μm sections were cut from paraffin embedded brains. Sections were collected onto Superfrost Plus slides (25 × 75 × 1 mm; Thermo Fisher Scientific, Massachusetts, United States). Paraffin-embedded brains underwent heat-mediated antigen retrieval using a citric acid buffer (0.21% citric acid and 0.05% Tween-20), then non-specific binding was blocked on both paraffin- and OCT-embedded brains by incubation with Normal Donkey Serum for 1 h. The tissue was incubated with antibodies against ionized calcium binding adaptor molecule 1 (IBA-1; goat polyclonal, AB5076, 1:500, Abcam, Cambridge, United Kingdom), Glial Fibrillary Acidic Protein (GFAP; rabbit polyclonal, Z0334, 1:1000, Agilent, California, United States) or CD68 (rat polyclonal, AB53444, 1:500, Abcam) at 4°C overnight. Subsequently, secondary donkey anti-goat Alexa Fluor 488 antibody (1:250; Invitrogen, Massachusetts, United States), donkey anti-rat Alexa Fluor 488 antibody (1:250; Invitrogen), or donkey anti-rabbit Alexa Fluor 594 antibody (1:250; Invitrogen) were applied and incubated for 1 h at room temperature. Sections were then counterstained with Hoechst (1:1000; Sigma-Aldrich, Missouri, United States) and mounted with glass coverslips using fluorescence mounting media (Agilent).

All fluorescent images were captured using the Nikon-TiE inverted fluorescence microscope with NIS-Elements (Nikon) software. Images were analyzed using FIJI/ImageJ (ver. 1.52p, National Institutes of Health, Maryland, United States). IBA-1, GFAP, and CD68 stains were quantified by converting the image to binary and the threshold was adjusted to best represent the original photomicrograph. The amount of staining was expressed as percentage coverage for each region of interest (Sharma et al., 2022). Image quantification methods were identical across all images. Every 6th section (~ 360 μm between sections) was analyzed from each brain, for a total of 8 slides between Bregma 0.7 to 3.5 mm.

### Microglia morphology analysis

Analysis of microglial morphology at 1-week post-injury was conducted as described previously (Ryu et al., 2021). In brief, five randomly selected IBA-1+ microglia were isolated from the three most medial images of the cortex of each brain at the acute time-point. Cell branches and soma were traced and skeletonized using ImageJ. The number of branches, average branch length and soma area were measured for each cell, then the average of all cells for each animal were calculated for graphical presentation.

Chronic microglial morphology was analyzed as previously published (Morrison et al., 2017; Young and Morrison, 2018; Green et al., 2022a,c). Three randomly selected microglia were isolated from the ipsilateral hippocampus. The three most medial sections were used from each animal; nine cells selected in total for each brain. Microglia were skeletonized and analyzed in ImageJ using the ‘analyze skeleton’ plugin. The number of branches, number of endpoints, and average branch length per cell were calculated.

### Cresyl violet staining

Coronal sections were stained with cresyl violet (Sigma-Aldrich, Missouri, United States), then dehydrated with increasing ethanol concentration, and mounted with DPX mounting medium (Sigma-Aldrich). Images were captured using the Leica Aperio AT Turbo Brightfield slide scanner at 1x magnification (Monash Histology Platform, Melbourne, Australia). The volume of pathological tissue was quantified using the Cavalieri method of unbiased stereology *via* ImageJ (Marcos et al., 2012; Sharma et al., 2022). The volume of remaining healthy tissue in the ipsilateral hemisphere was compared to the volume of healthy tissue in the contralateral hemisphere for each respective region.

### Quantitative real-time polymerase chain reaction

Fresh brains were collected from SHIP-1+/− and SHIP-1−/− mice at 6-h, 1-week and 12-weeks post-sham/TBI, for PCR to evaluate inflammatory gene expression. The ipsilateral cortex was processed for RNA isolation using a RNeasy mini kit (Qiagen, Hilden, Germany) and a QIAcube (Qiagen), as per the manufacturer’s protocol. Eluted RNA concentration and purity were determined using a Qiagen QIAexpert spectrophotometer. One microgram of eluted RNA from each sample was reversed transcribed into cDNA using QuantiTect Reverse Transcription kit (Qiagen) and diluted 1:10.

PCR for samples collected at 6-h and 1-week post-injury was run in duplicate on a 192-well plate using a Qiagility liquid handling robot (Qiagen). cDNA was amplified with the Vii7 Real-Time PCR System (Life Technologies, California, United States). Samples collected at 12-weeks post-injury were sent to the Monash Single Cell Genomics Facility (Monash Healthy Translation Precinct, Melbourne, Australia) for more extensive gene expression profiling. TaqMan fast advanced gene expression assay (Thermo Fisher Scientific) was used for both methods (Supplementary Table S3).

Relative gene expression ratios were calculated using the 2^−ΔΔCT^ method and normalized to the geometric mean of the housekeeping genes (*Ppia* and *Hprt*), as previously shown to be stable in this pediatric TBI model (Zamani et al., 2020). Expression of each gene was normalized to sham SHIP-1+/− control mice.

### Serum cytokine quantification

Serum cytokines at 12-weeks post-injury were quantified using the Bio-Plex Pro Mouse Cytokine 23-plex Assay (Bio-RAD, CA, United States) for IL-4, IL-6, IL10, IL-12(p40), G-CSF, IFN-γ, MIP-1α, MIP-1β Serum was diluted by 1:4, and 25 μL was loaded into each well. The plate was prepared as per the manufacturer’s instruction and washed using the Bio-Plex Handheld Magnetic Washer (Bio-RAD). The assays were run on the Bio-Plex 200 (Bio-RAD) and fluorescence intensity was collected. The calibration curve was analyzed using the Bio-Plex manager software (version 5.0, Bio-RAD).

### Statistical analysis

Statistical analysis was performed using the GraphPad Prism program (ver. 8.2.1, GraphPad Software, Boston, United States) with statistical significance reported as *p* < 0.05. All quantitative data were normally distributed based on normality tests (Anderson-Darling, D’Agostino and Pearson, Shapiro–Wilk and Kolmogorov–Smirnov); thus, two-way ANOVA with Tukey’s multiple comparison tests were used to examine the factors of genotype and injury. Where appropriate, three-way ANOVA tests were used to examine the factors of genotype, injury, and time. In addition, while the study was not designed to evaluate sex as a biological variable, we conducted three-way ANOVA tests as a preliminary analysis of sex in the context of genotype and injury variables; significant differences are reported. Data are presented as mean ± standard error of the mean (SEM).

## Results

### Inflammatory gene expression in the ipsilateral cortex was elevated at 6-h post-injury but not influenced by SHIP-1 deficiency

Mice were subjected to CCI or sham surgery at 3-weeks of age and assessed at several post-injury time points to determine the effect of SHIP-1 deficiency on a range of molecular, cellular, and neurobehavioral outcomes (Figure 1A). Gene expression in the ipsilateral cortex (Figure 1 and Supplementary Table S4) revealed increases in expression of the pro-inflammatory cytokine *Tnf* and chemokine *Ccl2* at 6-h post-injury in TBI animals (Figures 1B,C), while levels of *Il1b* were not changed (Figure 1D). In addition, at 6-h post-injury, TBI and SHIP-1 deficiency independently increased expression of the anti-inflammatory gene *Chil3* (Figure 1E). TBI alone increased expression of *H2ab1* and the astrogliosis marker *Gfap* (Figures 1F,G). Collectively, these studies indicate that TBI alone induced increased expression of inflammatory and neuroimmunological genes at 6 h post-injury but there was no difference between SHIP-1−/− mice and SHIP-1+/− mice.

### Microglial numbers and activation were not affected by genotype at 1-week following brain injury

Previous characterization of SHIP-1-deficient mice revealed impaired growth and splenomegaly manifestation as early as 4–5 weeks of age (Helgason et al., 1998). To confirm that our animals displayed a similar phenotype, we examined body weight and spleen weight. At 1-week post-injury (i.e., age p. 28), body weight was not affected by SHIP-1 deficiency or TBI (Supplementary Figure S2A). However, body weight was influenced by sex, specifically in TBI mice - where female SHIP-1−/− mice had a lower body weight compared to male SHIP-1−/− mice (Tukey’s multiple comparison test, *p* = 0.0176). SHIP-1−/− mice exhibited enlarged spleens (expressed as a ratio of body weight) compared to SHIP-1+/− mice, regardless of injury (Supplementary Figure S2B).

**Figure 2:**
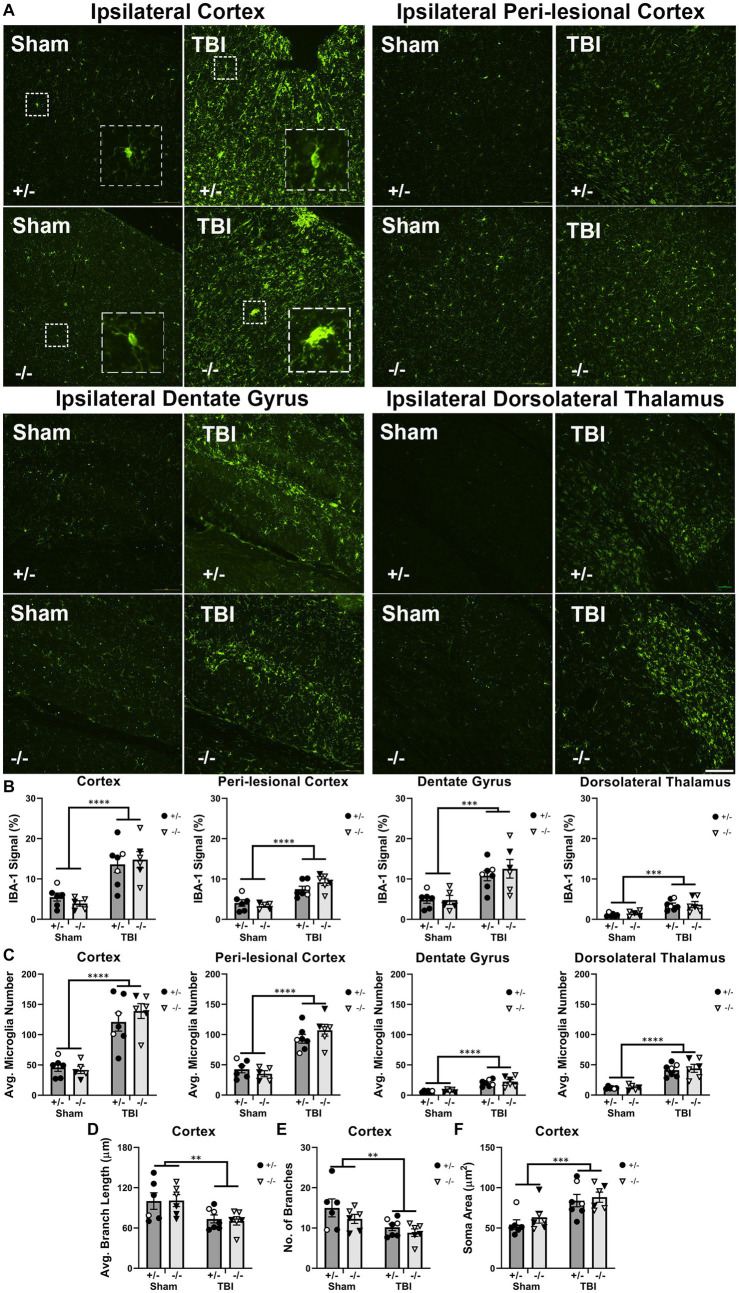
Microglial activation, number, and morphology in regions of interest were not affected by SHIP-1 deficiency acutely following TBI. **(A)** Representative images of IBA-1 immunofluorescence staining in the ipsilateral cortex, perilesional cortex, dentate gyrus and dorsolateral thalamus of SHIP-1+/− and SHIP-1−/− mice 1-week post-TBI or sham surgery. **(B,C)** Quantification of IBA-1 staining and the average number of IBA-1+ microglia in aforementioned brain regions of indicated mice. **(D–F)** Morphological analysis of ipsilateral cortical IBA-1+ microglia. **p < 0.01 ***p < 0.001 ****p < 0.0001, Two-way ANOVA main effect of injury; *n* = 5-7/group. Solid = female, open = male. Scale bar = 100 μm. Complete reporting of statistical analyzes is in Supplementary Table S5.

To investigate how SHIP-1 deficiency affected microglial activation at 1-week post-TBI, immunofluorescence staining was performed to quantify the expression of the microglial marker IBA-1, average number of IBA-1+ microglia, and their morphology (Figure 2 and Supplementary Table S5). The extent of IBA-1 staining and average number of IBA-1+ microglia were significantly increased at the injury site, peri-lesional area, dentate gyrus, and dorsolateral thalamus of TBI mice when compared with sham-treated mice (Figures 2B,C). However, there were no differences between genotypes in any of the four regions of interest.

In addition, the morphology of IBA-1+ microglia was analyzed as an indicator of cell activation state. A total of 15 cells were examined from each animal and the averages of each animal were compared. Compared to sham animals, microglia in the cortex of TBI animals exhibited reduced branch length and number of branches, indicating a more activated state was induced by TBI. Microglial activation was further indicated by increased average microglial cell soma size in TBI animals (Figures 2D–F). However, there were no differences in branch length, branch number, or soma size between genotypes. As expected, there were no TBI or genotype-driven effects in any microglial morphology outcome measures in the contralateral hemisphere regions-of-interest (data not shown). Taken together, the acute microglial activation following pediatric TBI was unaltered by SHIP-1 deficiency as IBA-1 expression, number of microglia and microglial morphology were comparable between SHIP-1−/− and SHIP-1+/− mice.

### Microglia-related gene expression was altered by SHIP-1 deficiency, but not TBI, at 1-week post-injury

**Figure 3:**
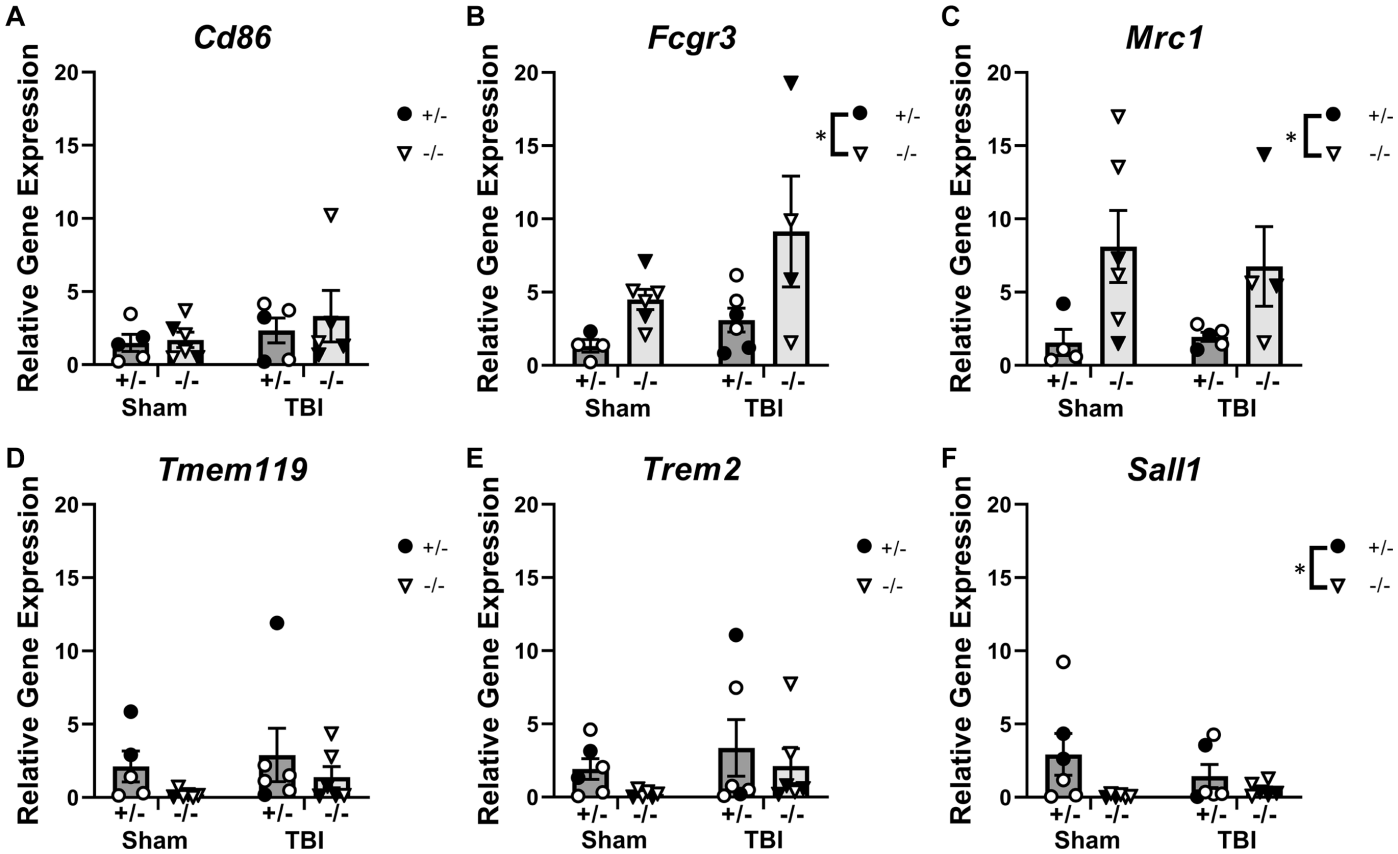
Acutely post-TBI, the expression of several microglial-related genes was altered by SHIP-1 deficiency. **(A–F)** Relative expression of the indicated genes in the injured cortex of SHIP-1+/− and SHIP-1−/− mice at 1-week post-injury. *p < 0.05, Two-way ANOVA main effect of genotype; *n* = 5-6/group. Solid = female, open = male. Complete reporting of statistical analyzes is in Supplementary Table S6.

To examine immune responses post-injury, expression of microglial-related inflammatory genes in the injured cortex was quantified at 1-week post-injury by PCR (Figure 3 and Supplementary Table S6). Expression of inflammatory markers such as *Cd86*, *Fcgr3* and *Mrc1* were unaffected by injury (Figures 3A–C). However, SHIP-1−/− mice exhibited increased gene expression of *Fcgr3* and *Mrc1* (Figures 3B,C). Similarly, Expression of the microglial transmembrane protein *Tmem119*, pattern recognition receptor *Trem2*, and microglial homeostatic marker *Sall1* were comparable between injury and sham groups (Figures 3D–F). However, SHIP-1−/− mice had decreased gene expression of *Sall1* compared to SHIP-1+/− mice (Figure 3F). Taken together, the absence of SHIP-1, but not TBI, altered the expression of inflammatory and immunological genes in 4-week-old mice.

### Ship-1 deficiency did not alter acute macrophage and astrocyte responses after TBI

**Figure 4:**
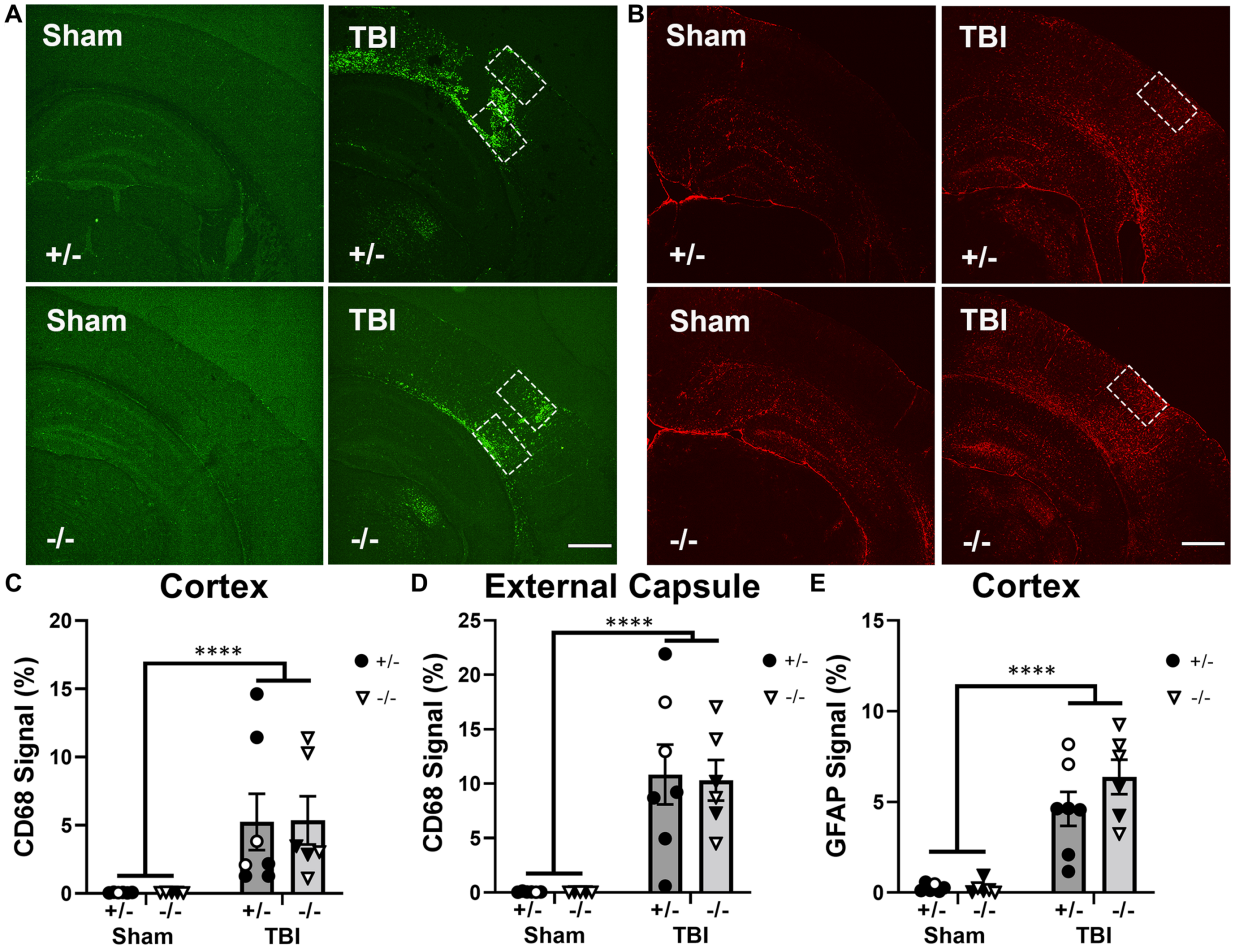
SHIP-1 deficiency did not alter acute CD68 and GFAP expression at 1-week after TBI. **(A)** Representative images of CD68 (green) and **(B)** GFAP (red) immunofluorescence staining in the ipsilateral dorsal hemisphere of sham and TBI SHIP-1+/− and SHIP-1−/− mice 1-week post injury. Quantification of CD68 staining at the **(C)** injury site and **(D)** external capsule, and quantification of GFAP staining at the **(E)** injury site. **p < 0.01 ****p < 0.0001, Two-way ANOVA main effect of injury; *n* = 5-7/group. Solid = female, open = male. Scale bar = 1 mm. Complete reporting of statistical analyzes is in Supplementary Table S7.

In addition to microglial responses, innate immune responses in the brain including macrophage infiltration (by CD68 immunofluorescence) and astrocyte reactivity (by GFAP immunofluorescence) were also evaluated at 1-week post-injury in SHIP-1+/− and SHIP-1−/− mice (Figure 4 and Supplementary Table S7).

CD68 immunofluorescence staining at the injury site and ipsilateral corpus callosum/external capsule was examined to quantify macrophage infiltration, as previously observed (Huang et al., 2021). While CD68 can label both microglia and macrophages, we interpreted this as predominantly macrophages in this context as expression appeared to be restricted to amoeboid-like cells in close proximity to the lesion site. CD68 immunofluorescence staining was significantly increased in both SHIP-1+/− and SHIP-1−/− mice after TBI compared to sham animals (Figures 4C,D); however, there were no differences in CD68 staining between genotypes within both regions. Expression of *Ccl2*, a gene encoding a chemokine central to monocyte recruitment, was quantified at the injury site, and found to be elevated after injury (Supplementary Figure S3), but not affected by SHIP-1 deficiency. Similarly, GFAP immunofluorescence was increased in TBI animals at this acute time-point, but no differences were observed between genotypes (Figure 4E). This indicates that macrophage infiltration and astrocyte reactivity acutely post-injury were not affected by the absence of SHIP-1.

### Ship-1 deficiency did not alter TBI-induced tissue damage or changes in anxiety-like behavior, explorative tendency, and memory functioning at 1-week post-injury

**Figure 5:**
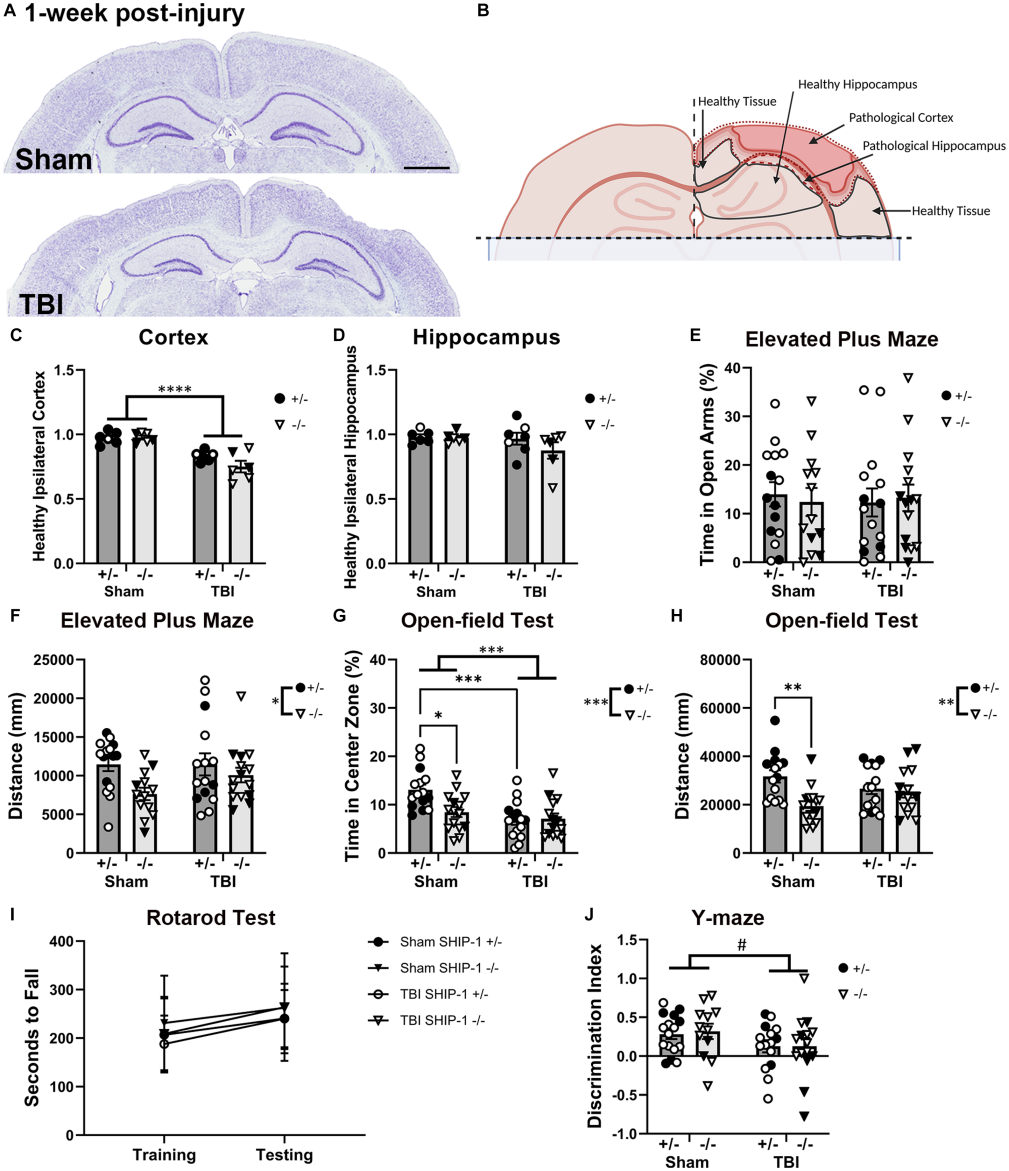
SHIP-1 deficiency induced anxiety-like behavior and reduced explorative tendency at 1-week post-injury, without altering the extent of brain tissue damage. **(A)** Representative images of brain sections from sham and TBI SHIP-1+/− stained with cresyl violet at 1-week post-injury. **(B)** Illustration of regions of interest. **(C)** Ratio of healthy (remaining/uninjured) ipsilateral cortex volume to healthy contralateral cortex volume. **(D)** Volume of healthy ipsilateral hippocampus to healthy contralateral hippocampus. **(E)** Percentage time spent in open arm and **(F)** total distance traveled during the Elevated Plus Maze. **(G)** Percentage time spent in the center zone and **(H)** total distance traveled during the open-field test. **(I)** Latency to fall during the rotarod test on the training and testing days. **(J)** Comparison between time spent in familiar arm and novel arm during the Y-maze. *p < 0.05 **p < 0.01 ***p < 0.005 ****p < 0.0001; Two-way or three-way ANOVA with Tukey’s post-hoc main effect of injury and genotype (and time, as appropriate); *n* = 5-17/group. Solid = female, open = male. Scale bar = 2 mm. Complete reporting of statistical analyzes is in Supplementary Table S8.

Cresyl violet staining of brain tissue sections was used to examine the extent of tissue damage after TBI (Figure 5 and Supplementary Table S8). The remaining volume of healthy tissue in the ipsilateral cortex and hippocampus were compared to the volume in the contralateral hemisphere (Figure 5B). In the ipsilateral hemisphere, the volume of remaining healthy cortical tissue was reduced at 1-week post-injury, independent of genotype (Figure 5C), while the volume of healthy tissue in the ipsilateral hippocampus at 1-week post-injury was not affected by either injury or genotype (Figure 5D). The acute pathological outcomes in this study were consistent with the progressive pathology as previously described (Webster et al., 2019b; Sharma et al., 2022, 2023).

SHIP-1+/− and SHIP-1−/− mice also underwent a battery of behavior tests prior to tissue collection at 1-week post-injury, to examine whether SHIP-1 deficiency alters TBI-induced behavioral deficits commonly reported in this pediatric CCI model (Webster et al., 2019a; Sharma et al., 2022). In the Elevated Plus Maze, although the amount of time in the open arms was comparable between genotypes and injury groups (Figure 5E), the total distance traveled by SHIP-1−/− mice was lower (Figure 5F).

In the open-field test, both SHIP-1 deficiency and TBI resulted in decreased time spent in the center zone (Figure 5G). Post-hoc testing indicated reduced time in center in sham-operated SHIP-1−/− mice compared to sham operated SHIP-1+/− mice (Tukey’s multiple comparison test, *p* = 0.0115), as well as, reduced time in center in SHIP-1+/− mice with TBI compared to sham-treated SHIP-1+/− mice (Tukey’s multiple comparison test, *p* = 0.0004; Figure 5G). However, there were no differences between SHIP-1+/− and SHIP-1−/− mice post-injury.

Consistent with findings from the Elevated Plus Maze, SHIP-1−/− mice also displayed reduced explorative tendencies during the open-field test as determined by distance traveled (Figure 5H). Furthermore, preliminary analysis revealed an influence by sex, as distance traveled by sham-treated male mice was lower compared to sham-treated female mice (Tukey’s multiple comparison test, *p* = 0.0334; Figure 5H). This reduced activity observed in SHIP-1−/− mice was not due to general motor deficits in these animals, as the performance during the accelerating rotarod task was unaffected by both injury and genotype (Figure 5I). Additionally, all groups regardless of injury or genotype showed an increase in rotarod performance on the test day compared to the training day (Figure 5I).

In the Y-maze, the discrimination index was calculated to examine each animal’s short-term spatial memory performance and ability to discern the familiar arm from the novel arms of the maze. Performance during Y-maze was comparable between injury groups and genotype (Figure 5J). Preliminary assessment revealed an interaction between sex and genotype (Figure 5J).

In summary, SHIP-1 deficiency did not exacerbate tissue loss 1-week following TBI, but independently increased anxiety-like behaviors and reduced the explorative tendency. Neither TBI nor SHIP-1 deficiency affected motor skills or working memory at 1-week post-injury.

### At 12-weeks post pediatric TBI, SHIP-1 deficient mice had exacerbated body weight differences, splenomegaly, and serum cytokine levels

**Figure 6:**
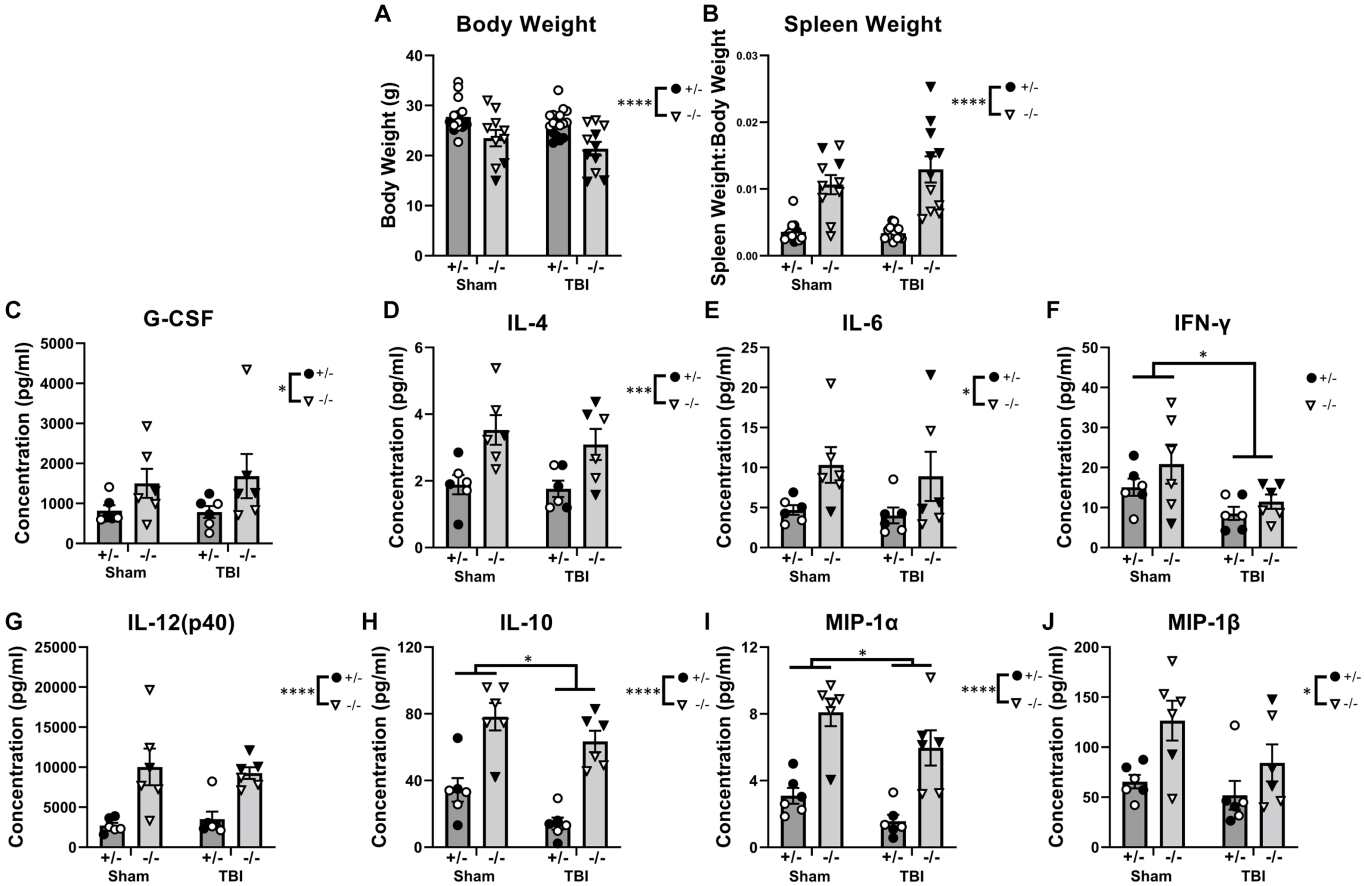
SHIP-1 deficiency led to reduced body weight, splenomegaly, and elevated serum cytokines at 12-weeks post-injury. **(A)** Body weights of SHIP-1+/− and SHIP-1−/− mice 12-weeks post-TBI or sham surgery. **(B)** Spleen weight expressed relative to body weight. **(C–J)** Concentration of the indicated cytokines in the serum of aforementioned mice. *p < 0.05, **p < 0.01, ***p < 0.005, ****p < 0.0001, Two-way ANOVA main effect of injury and genotype; **(A,B)** *n* = 10-18/group, **(C–J)** *n* = 5-6/group. Solid = female, open = male. Complete reporting of statistical analyzes is in Supplementary Table S9.

Due to pro-longed secondary complications following pediatric TBI clinically (Neumane et al., 2021), injury outcomes and immunological responses were also assessed chronically post-injury (12-weeks). Body weight, spleen weight, and serum cytokines were examined as a measure of the peripheral inflammatory phenotype (Figure 6 and Supplementary Table S9). At 12-weeks post-injury, SHIP-1−/− mice had reduced body weight and exhibited splenomegaly, independent of injury, likely reflecting their known peripheral inflammatory phenotype (Figures 6A,B; Helgason et al., 1998; Liu et al., 1999; Maxwell et al., 2011; Tsantikos et al., 2018). Moreover, sex had an impact on body weight and spleen weight. Preliminary post-hoc assessment identified a reduction in body weight of sham-treated female SHIP-1−/− mice (Figure 6A, Tukey’s multiple comparisons test, *p* = 0.0375), whereas spleen weight was elevated post-TBI in female SHIP-1−/− mice (Figure 6B, Tukey’s multiple comparisons test, *p* < 0.0001).

In addition, SHIP-1−/− mice had elevated circulating cytokines including G-CSF, IL-4, and IL-6 compared to SHIP-1+/− mice, with no differences between sham and injury groups, which is consistent with previous studies (Figures 6C–E; Desponts et al., 2006; Haddon et al., 2009; Maxwell et al., 2011; Tsantikos et al., 2018). By contrast, the concentration of the pro-inflammatory cytokine IFN-γ was significantly reduced in TBI mice, whereas IL-12(p40) levels were significantly higher in SHIP-1−/− mice (Figures 6F,G). The anti-inflammatory cytokine IL-10 and chemoattractant MIP-1α showed main effects of both genotype and TBI, being significantly reduced in TBI animals but increased in SHIP-1−/− mice (Figures 6H,I). Finally, the concentration of MIP-1β was also significantly elevated in SHIP-1−/− mice, in both TBI and sham groups (Figure 6J). Together these data indicate that absence of SHIP-1 elevated circulating cytokine concentrations, while TBI had an immunosuppressive effect chronically post-pediatric brain injury.

### Inflammatory gene expression was elevated in the hippocampus of SHIP-1-deficient mice chronically after TBI

**Figure 7:**
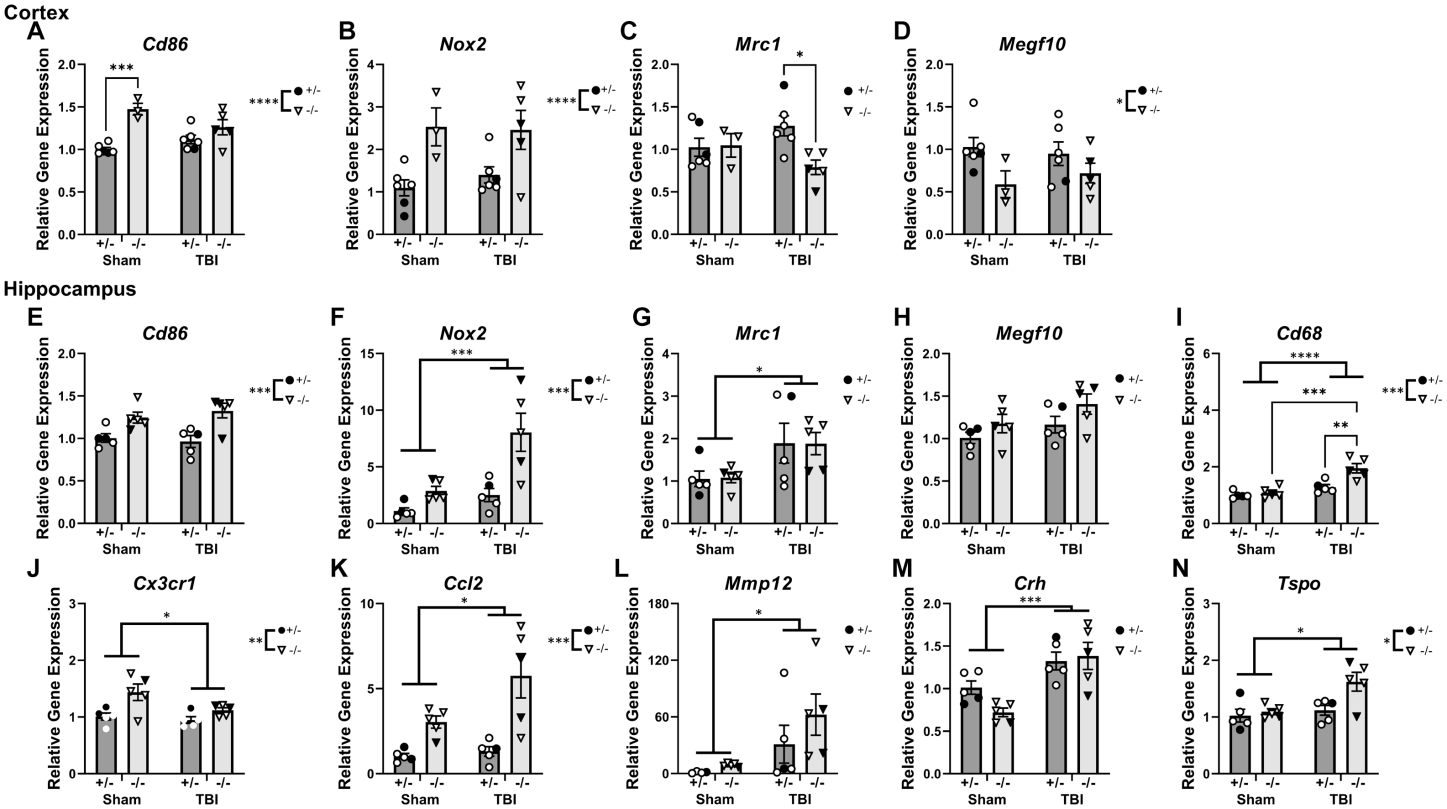
Interactions were observed between SHIP-1 deficiency and TBI in inflammatory gene and microglial phagocytic gene expression chronically post-injury, particularly in the hippocampus. **(A–D)** Relative expression of the indicated genes in the ipsilateral cortex of SHIP-1+/− and SHIP-1−/− mice at 12-weeks post-injury. **(E–N)** Relative expression of the indicated genes in the ipsilateral hippocampus of SHIP-1+/− and SHIP-1−/− mice at 12-weeks post-injury. *p < 0.05, **p < 0.01, ***p < 0.005, ****p < 0.0001; Two-way ANOVA with Tukey’s post-hoc main effects of injury and genotype; *n* = 3-6/group. Solid = female, open = male. Complete reporting of statistical analyzes is in Supplementary Table S10.

To examine whether chronic immune responses post-injury was exacerbated by SHIP-1 deficiency, PCR was performed to examine the expression of genes related to inflammatory and immune responses in the ipsilateral cortex and hippocampus of SHIP-1+/− and SHIP-1−/− mice chronically post-TBI (Figure 7 and Supplementary Table S10). Quantification of *Inpp5d* expression in the cortex confirmed a lack of SHIP-1 expression in SHIP-1−/− mice (Supplementary Figure S4). In the ipsilateral cortex, expression of the pro-inflammatory genes *Cd86* elevated in SHIP-1−/− mice, with post-hoc analysis identifying increased expression in sham operated SHIP-1−/− mice compared to similarly treated SHIP-1+/− mice (Tukey’s multiple comparisons test, *p* = 0.0006; Figure 7A). *Nox2* expression was elevated in SHIP-1−/− mice (Figure 7B). Interactions between injury and SHIP-1 deficiency decreased the expression of the anti-inflammatory marker *Mrc1*, with post-hoc analysis revealing a significant reduction in SHIP-1−/− mice compared to SHIP-1+/− mice in the TBI group (Tukey’s multiple comparisons test, *p* = 0.0257; Figure 7C). Additionally, expression of the astrocyte activation marker *Megf10* was unchanged between injury groups, but significantly lower in SHIP-1−/− mice compared to SHIP-1+/− mice (Figure 7D). Taken together, these results suggest that the expression of inflammatory genes in the cortex was altered by SHIP-1 deficiency at 12 weeks post-injury.

In the ipsilateral hippocampus, *Cd86* expression was comparable between injury groups, but was significantly elevated in SHIP-1−/− mice compared to SHIP-1+/− mice (Figure 7E). *Nox2* expression in the hippocampus was elevated in both SHIP-1 deficient mice and TBI mice, with a trend towards a significant interaction between SHIP-1 deficiency and injury (Figure 7F). Moreover, injury alone elevated *Mrc1* expression in the hippocampus, with no differences between genotypes (Figure 7G). Expression of *Megf10* within the hippocampus was comparable between injury and genotypes (Figure 7H).

Hippocampal expression of the microglial phagocytic gene *Cd68* was elevated by both SHIP-1 deficiency and injury, with post-hoc analysis identifying elevated expression in SHIP-1−/− mice after TBI, compared to SHIP-1+/− mice in the TBI group and SHIP-1−/− mice in the sham group (Tukey’s multiple comparisons test, *p* = 0.0031; *p* = 0.0055; Figure 7I). On the contrary, gene expression of the chemokine receptor *Cx3cr1* was reduced in the TBI mice, but overall elevated in SHIP-1−/− mice compared to SHIP-1+/− mice (Figure 7J).

Gene expression of the chemokine *Ccl2* in the hippocampus was increased following injury and SHIP-1 deficiency (Figure 7K). Moreover, gene expression of the blood–brain barrier breakdown marker *Mmp12* was elevated in the hippocampus of TBI mice, with no differences between genotypes (Figure 7L).

Expression of the stress hormone *Crh* was elevated in the hippocampus of TBI mice (Figure 7M). Finally, expression of the neuroinflammatory marker *Tspo*, was increased by both injury and SHIP-1 deficiency (Figure 7N). In summary, the expression of inflammatory genes in the hippocampus chronically post-injury were altered by SHIP-1 deficiency and injury.

Microglia did not exhibit an activated phenotype at 12-weeks post-pediatric TBI, despite an elevated astrocyte response.

**Figure 8:**
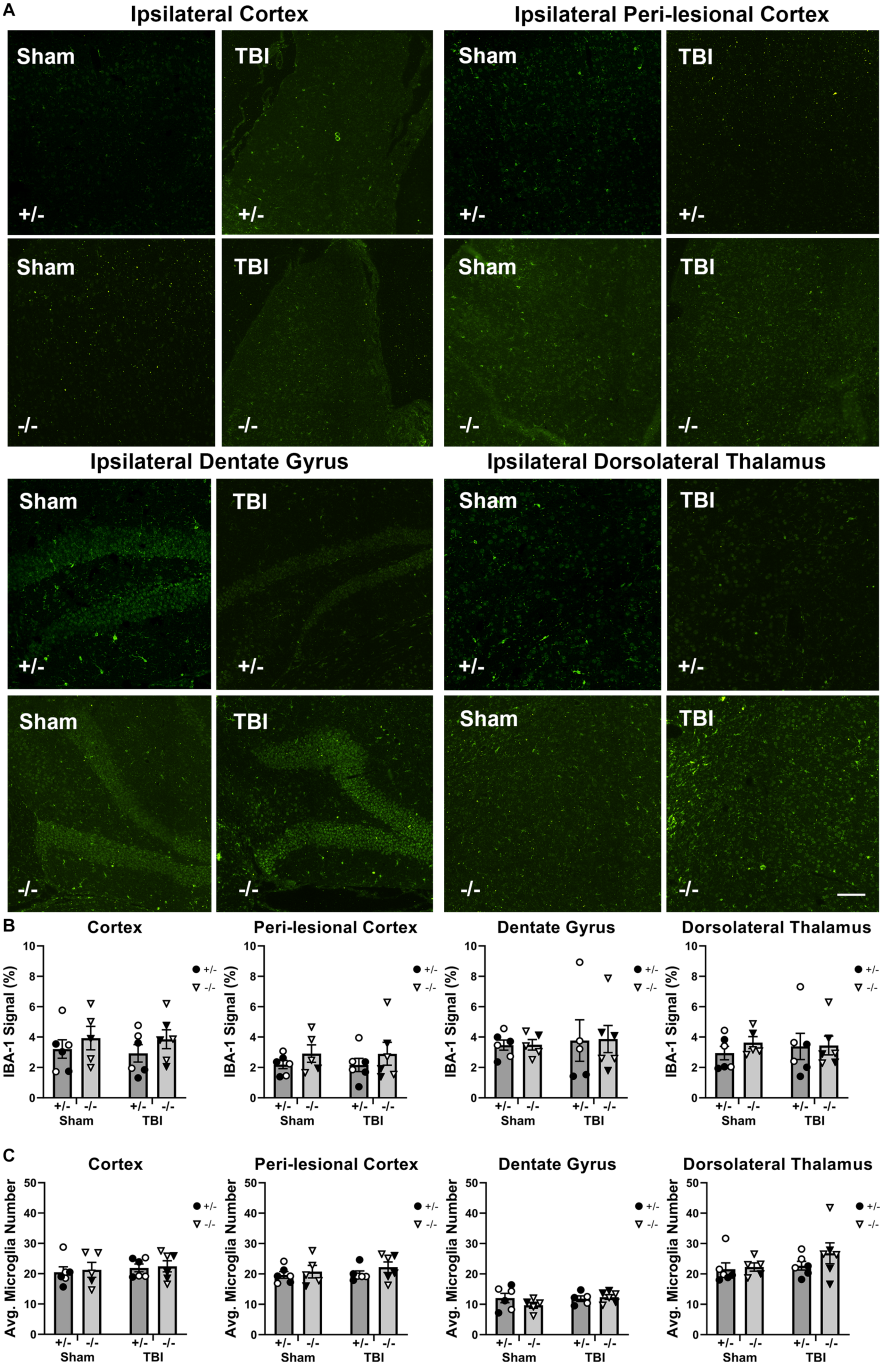
Chronic microglial activation was not altered by SHIP-1 deficiency or pediatric TBI. **(A)** Representative images of IBA-1 immunofluorescence staining of brain sections of SHIP-1+/− and SHIP-1−/− mice at 12-weeks post-injury. **(B)** IBA-1 expression in indicated regions of interest. **(C)** Number of IBA-1 positive cells in indicated regions of interest. Two-way ANOVA; *n* = 5-6/group. Solid = female, open = male. Scale bar = 100 μm. Complete reporting of statistical analyzes is in Supplementary Table S11.

Next, we examined the inhibitory effect of SHIP-1 in chronic glial responses by assessing microglia activation, morphology, and astrocyte reactivity in SHIP-1−/− mice at 12-weeks following pediatric brain injury, given prolonged glial activation following focal brain injuries was previously identified in pre-clinical studies (Loane et al., 2014; Figures 8 and Supplementary Table S11). At this chronic timepoint, IBA-1 immunofluorescence staining and the number of IBA-1+ microglia at the ipsilateral injury site, peri-lesional area, dentate gyrus and dorsolateral thalamus was comparable between injury groups and genotypes (Figures 8B,C). Additionally, there were no differences between groups in the extent of IBA-1 staining or number of IBA-1 positive microglia in the contralateral hemisphere (data not shown).

**Figure 9:**
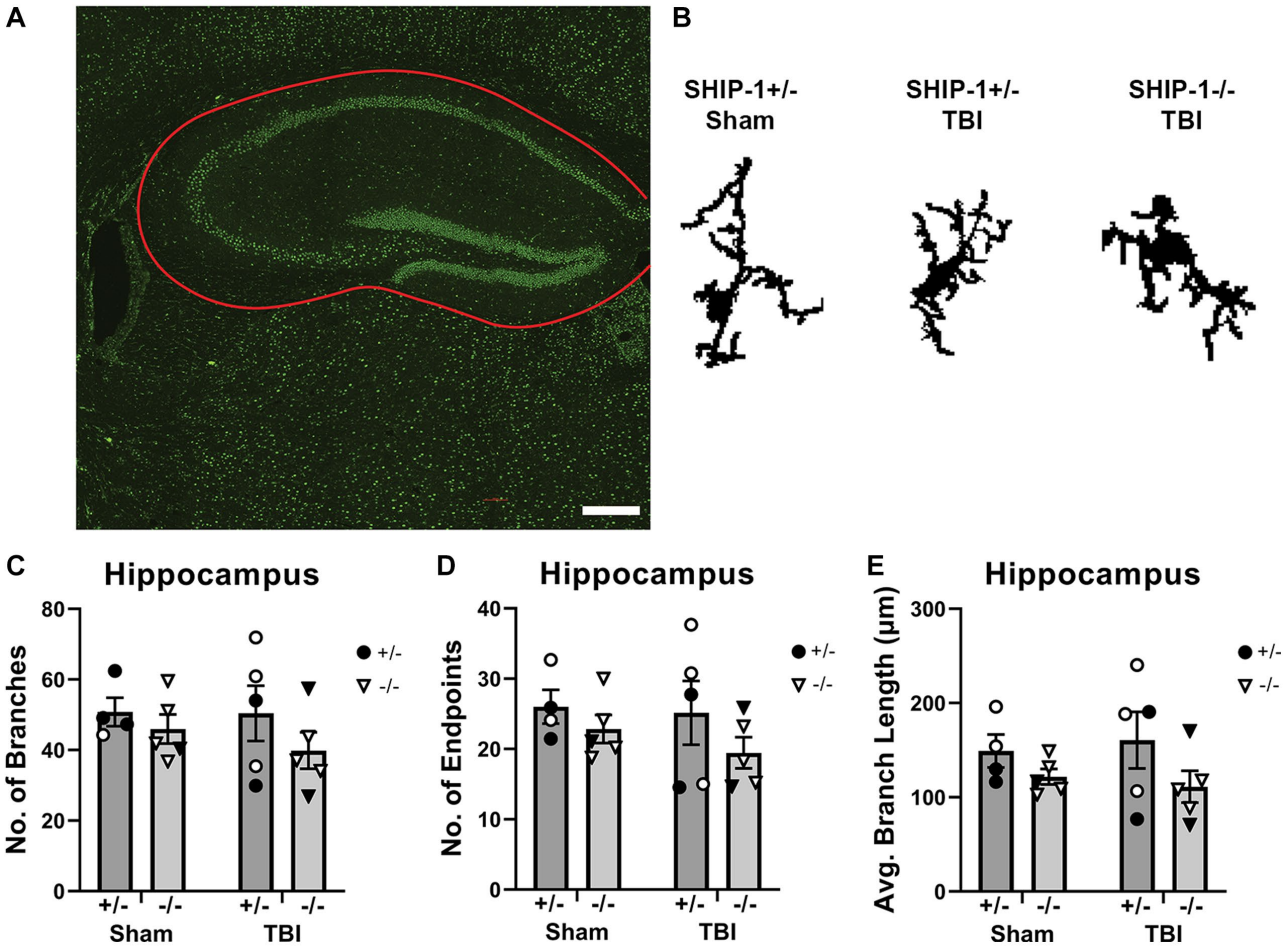
SHIP-1 deficiency and pediatric TBI did not alter chronic microglial morphology in the hippocampus. **(A)** Representative image of IBA-1 staining in the hippocampus **(B)** with three randomly selected microglia isolated from IBA-1-stained images of SHIP-1+/− and SHIP-1−/− mice at 12-weeks post-injury. The total number of **(C)** branches and **(D)** branch end points, and **(E)** average branch length was quantified by single cell morphological analysis. Two-way ANOVA; *n* = 4-6/group. Solid = female, open = male. Scale bar = 200 μm. Complete reporting of statistical analyzes is in Supplementary Table S11.

Since inflammatory gene expression was significantly elevated in the hippocampus at 12-weeks post-injury suggesting an increase in microglial activation, microglial morphology in the ipsilateral hippocampus were examined using single cell quantitative analysis (Figure 9 and Supplementary Table S11; Morrison et al., 2017; Green et al., 2022c). Three cells were isolated from each image of the hippocampus, from the three most medial images from each animal (Figure 9B). The average number of branches, number of branch end points, and average branch length were comparable between sham and injury groups and genotypes (Figures 9C–E).

**Figure 10:**
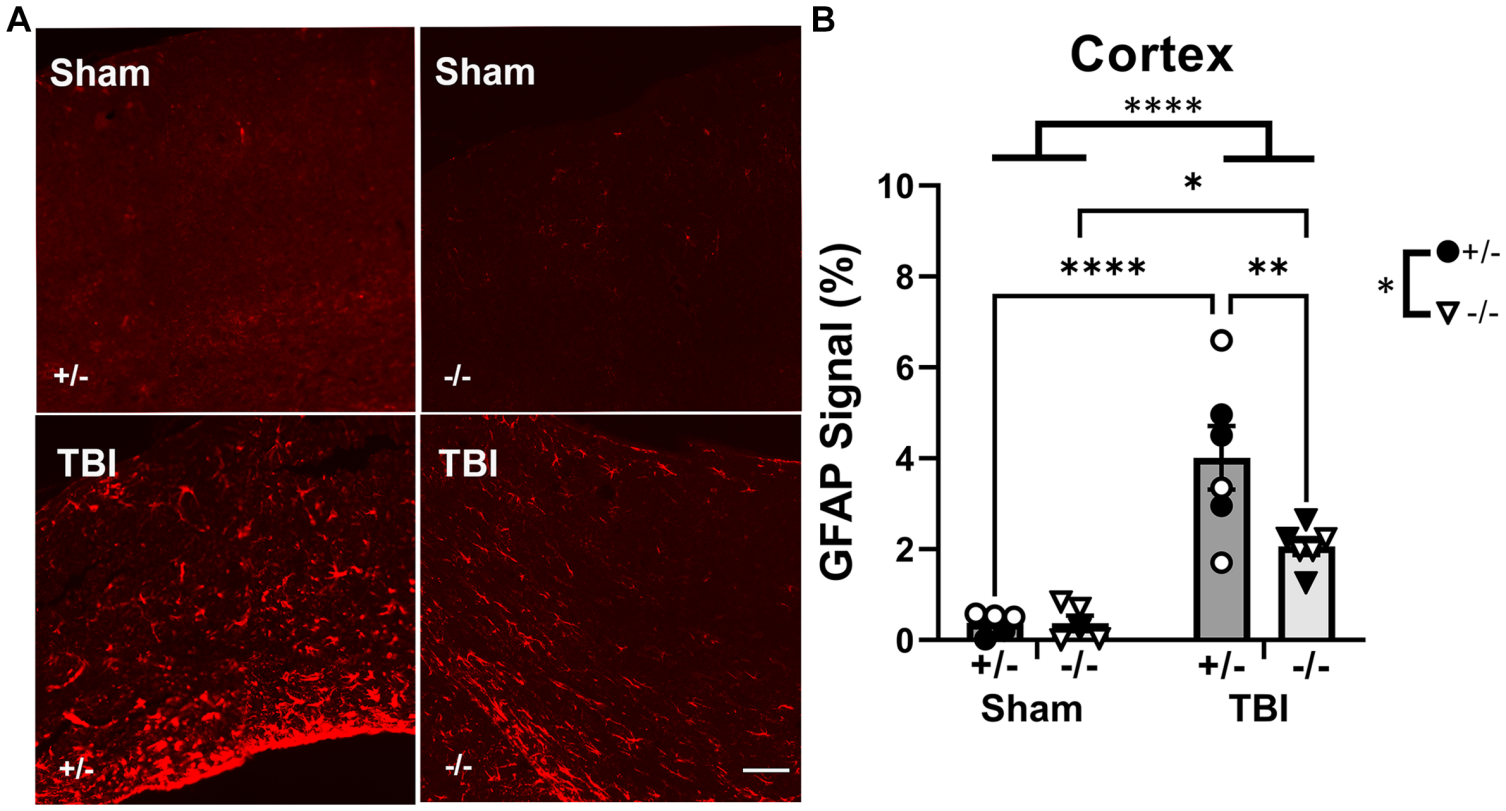
Chronic GFAP expression was reduced by SHIP-1 deficiency post-TBI. **(A)** Representative images of GFAP staining at the ipsilateral cortex of SHIP-1+/− and SHIP-1−/− animals at 12-weeks post-injury. **(B)** Quantitation of GFAP immunofluorescence staining of the cortex at 12-weeks post-injury. *p < 0.05, **p < 0.005, ****p < 0.0001. Two-way ANOVA with Tukey’s post-hoc; *n* = 5-6/group. Solid = female, open = male. Scale bar = 100 μm. Complete reporting of statistical analyzes is in Supplementary Table S11.

To quantify GFAP+ astrocyte reactivity, GFAP immunofluorescence staining in the ipsilateral cortex was increased after injury but reduced in SHIP-1−/− mice (Figure 10). Post-hoc analysis revealed a reduction in SHIP-1−/− mice post-TBI compared to similarly treated SHIP-1+/− mice (Tukey multiple comparisons test, *p* = 0.009; Figure 10). Nevertheless, GFAP staining in the dentate gyrus was not affected by injury or genotype at 12-weeks post-injury (data not shown). There were no differences in the amount of GFAP staining in the contralateral cortex and dentate gyrus (data not shown). In summary, chronic IBA-1 expression, number of microglia and microglial morphology were not altered by injury or SHIP-1 deficiency. However, chronic astrocyte reactivity remained elevated post-injury, but reduced in the absence of SHIP-1.

### SHIP-1 deficiency independently reduced explorative tendency and impaired working memory during early-adulthood

**Figure 11:**
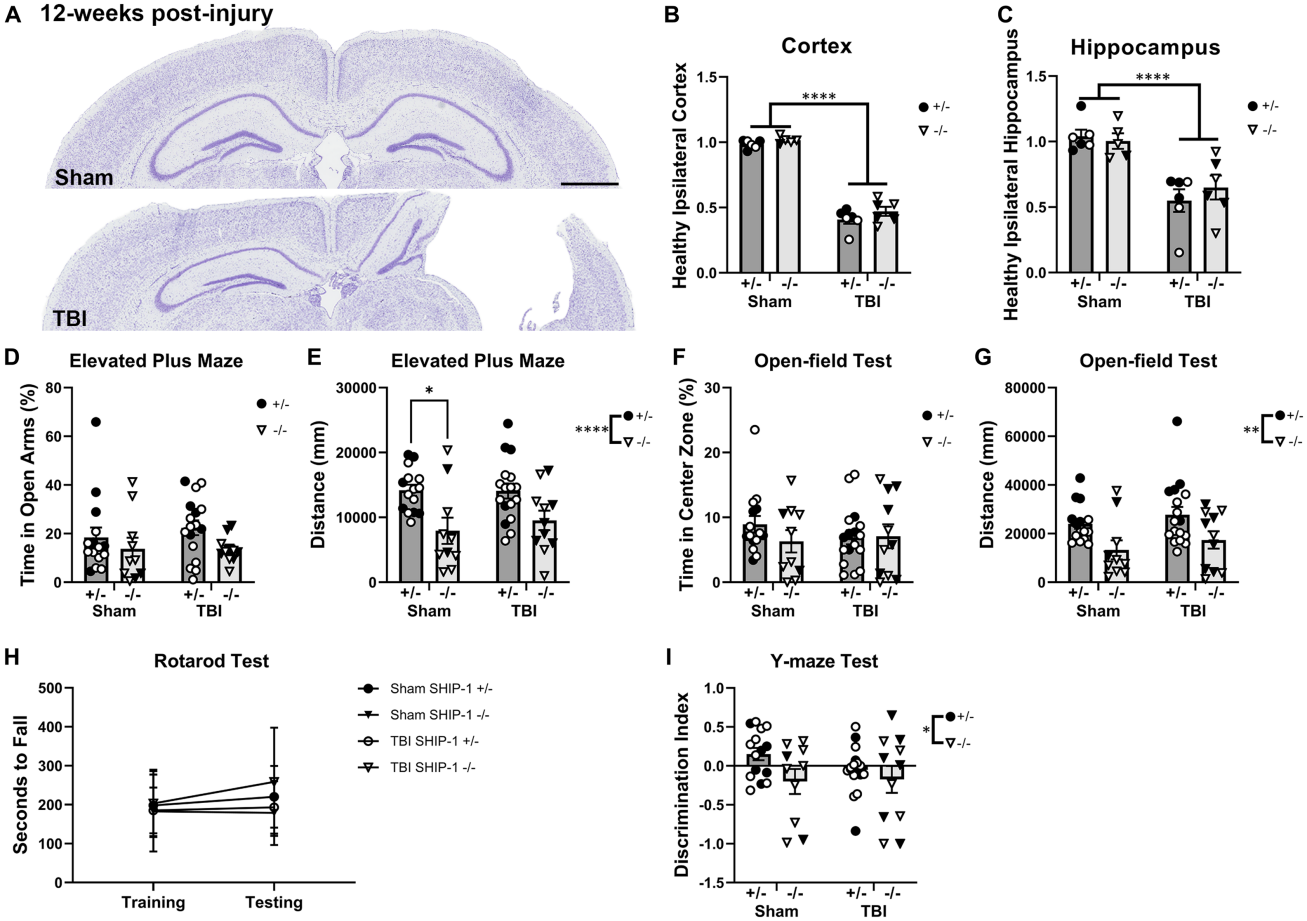
SHIP-1 deficiency did not alter the extent of tissue damage at 12 weeks post-injury but impacted general activity and working memory. **(A)** Representative images of cresyl violet-stained brain sections from sham and TBI SHIP-1+/− mice. Volume of **(B)** remaining healthy ipsilateral cortex, and **(C)** ipsilateral hippocampus. **(D)** Percentage time spent in open arms and **(E)** total distance traveled during Elevated Plus Maze. **(F)** Percentage time spent in the center zone and **(G)** total distance traveled during open-field test. **(H)** Latency to fall from the rotarod test. **(I)** Discrimination index calculated (amount of time spent in novel arm versus the familiar arm during the Y-maze). *p < 0.05, **p < 0.005, ****p < 0.0001, Two-way and three-way ANOVA; **(B,C)** *n* = 5-6/group, **(D–I)** *n* = 11-18/group for behavior. Solid = female, open = male. Scale bar = 1 mm. Complete reporting of statistical analyzes is in Supplementary Table S12.

To examine whether SHIP-1 deficiency affected tissue loss chronically post-injury, pediatric post-natal day 21 SHIP-1+/− and SHIP-1−/− mice underwent sham or CCI injury, then volumetric analysis was conducted to assess remaining healthy tissue at 12-weeks post-injury (Figure 11 and Supplementary Table S12). Both SHIP-1+/− and SHIP-1−/− mice exhibited a significant reduction in remaining healthy tissue in the ipsilateral cortex and hippocampus compared to sham mice (Figures 11A–C). However, there were no differences in tissue volume between SHIP-1+/− and SHIP-1−/− mice in either brain region (Figures 11A–C).

At 12-weeks post-injury, SHIP-1+/− and SHIP-1−/− mice underwent a battery of behavior tests prior to tissue collection to examine chronic behavioral changes following brain injury. Time spent in the open arms during the Elevated Plus Maze test was comparable between injury groups and genotypes (Figure 11D). However, SHIP-1−/− mice had reduced activity compared to control SHIP-1+/− mice as determined by distance traveled (Figure 11E).

The time spent in the center during the open-field test was comparable between injury groups and genotypes (Figure 11F). However, SHIP-1−/− mice exhibited reduced locomotor/exploratory activity as determined by distance traveled (Figure 11G). There were no differences in the rotarod test scores between injury groups or genotypes, and no improvements in their performance across both days (Figure 11H). Furthermore, preliminary assessment identified an influence of sex in SHIP-1+/− after TBI, as female mice traveled a greater distance during the open-field test compared to male mice (Figure 11H, Tukey multiple comparisons test, *p* = 0.0134).

Lastly, SHIP-1−/− mice showed poorer ability to discern the novel arm from the familiar arm in the Y-maze test when compared to SHIP-1+/− mice (Figure 11J). However, sham and TBI animals performed similarly at 12-weeks post-injury (Figure 11J). Together, these findings demonstrate that SHIP-1 is important for general exploratory/locomotor activity and cognition, but this phenotype was not exacerbated by a prior early-life TBI. Collectively, SHIP-1 deficiency did not impact chronic tissue loss at the cortex and hippocampus but led to reduced explorative tendency and impaired working memory at 12-weeks post-injury.

## Discussion

**Figure 12:**
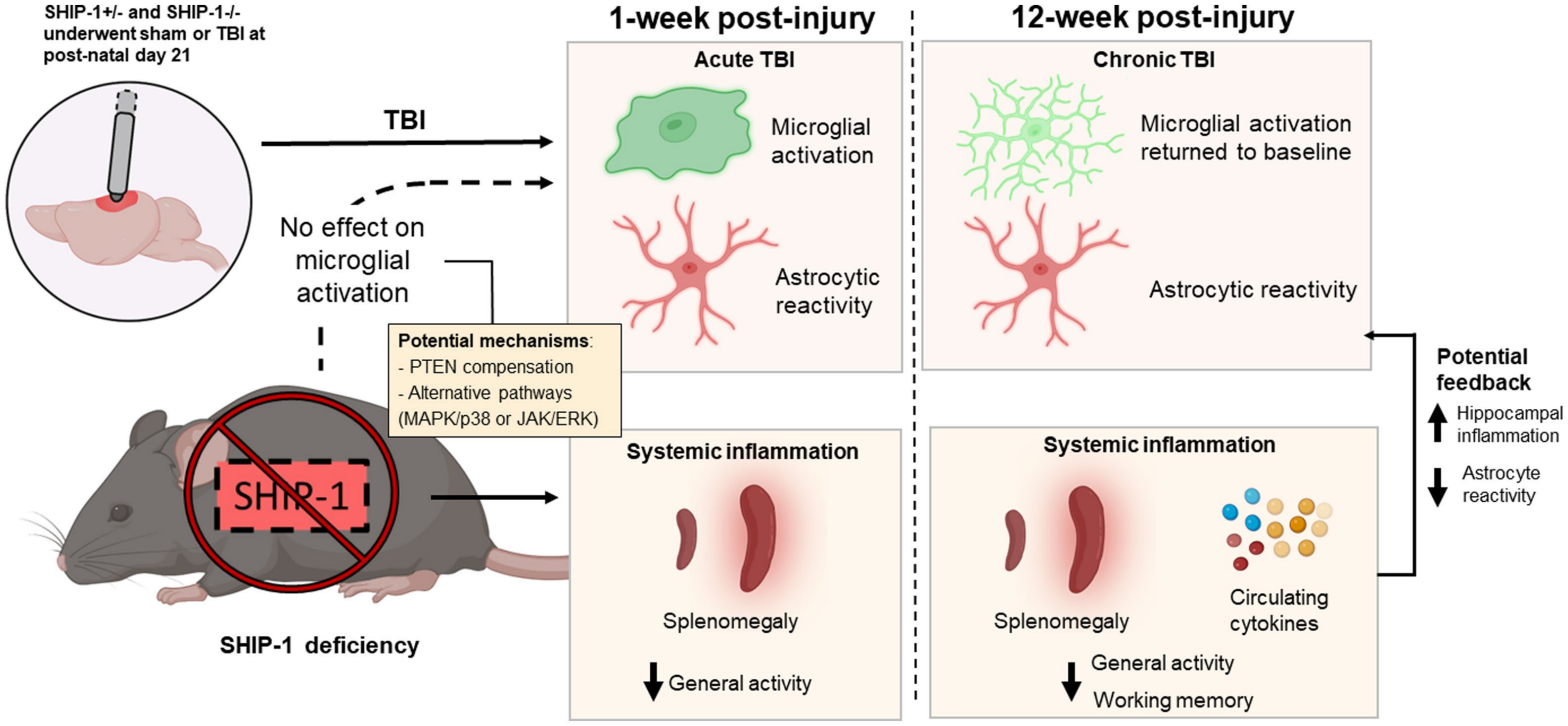
Graphical summary of the main findings in this study. SHIP-1+/− and SHIP-1−/− mice underwent sham or TBI surgeries at post-natal day 21. TBI alone induced microglial activation and astrocyte reactivity at 1-week post-injury. However, at 12-week post-injury, microglial activation had returned to baseline, but astrocyte reactivity remained. Global SHIP-1 deficiency had no effect on microglial activation, but based on previous findings, it likely resulted in systemic inflammation which may have impacted general activity and working memory. Chronic systemic inflammation may also exacerbate hippocampal inflammation and suppress astrocyte reactivity at 12-weeks post-injury.

Previous studies using gene deficient mice have revealed a critical regulatory role for SHIP-1 in immune cell signaling, with perturbation of the pathway resulting in inflammatory lung and gastrointestinal diseases (Helgason et al., 1998; Liu et al., 1999; Takeshita et al., 2002; Kerr et al., 2011; Maxwell et al., 2011; McLarren et al., 2011; Hibbs et al., 2018). While the expression of SHIP-1 in microglia has been reported, its role in regulating inflammatory immune responses in the brain has scarcely been explored to date (Zhang et al., 2014, 2016; Olah et al., 2018; Pedicone et al., 2020; Marsh et al., 2022). Here we evaluated the neuroimmunological, pathological and behavioral consequences of SHIP-1 deficiency after experimental pediatric TBI (Figure 12). We report that immune responses within the first week after a pediatric TBI occurred independently of SHIP-1, and that subsequent tissue damage and behavioral changes were predominantly TBI-driven. However, SHIP-1 deficiency resulted in exacerbated chronic inflammatory responses in the hippocampus following TBI, alongside unexpected suppression of astrocyte reactivity and a reduction of peripheral inflammatory cytokine levels (Figure 12).

Throughout, SHIP-1−/− mice were compared to SHIP-1+/− littermates as the control strain, in place of SHIP-1+/+ or traditional C57Bl/6 mice. While deletion of one allele may result in functional changes depending on the gene under investigation, we and others have previously conducted extensive evaluations to compare SHIP-1+/− and SHIP-1+/+ across a range of peripheral phenotypes and found them to be indistinguishable (see Supplementary Figure S1 and Supplementary Table S1; Maxwell et al., 2011; Anderson et al., 2015), justifying the use of SHIP-1+/− littermates as the preferred control strain in this study.

Immune responses are typically upregulated acutely following moderate to severe experimental TBI in rodents (Israelsson et al., 2009; Turtzo et al., 2014; Witcher et al., 2021), consistent with what is observed in patients after TBI (Lenzlinger et al., 2001; Lindblad et al., 2021; Shultz et al., 2022). We observed an acute increase in inflammatory gene expression (i.e., *Tnf* and *Ccl2*) in the ipsilateral cortex of both SHIP-1+/− and SHIP-1−/− mice. These changes in gene expression were transient, returning to sham levels by 1-week post-injury. While serum cytokine levels were not available at the acute time points post-injury for this study, it is expected that this would have further confirmed an acute inflammatory response (Woodcock and Morganti-Kossmann, 2013). Independent of TBI, elevated *Fcgr3* and *Mrc1* expression in the cortex of SHIP-1−/− mice suggests basal changes in microglial phagocytic ability in the absence of SHIP-1. Similar alterations have been previously described in SHIP-1-deficient macrophages (Marzolo et al., 1999; Kamen et al., 2008; Kigerl et al., 2009). Reduced *Sall1* expression in SHIP-1−/− microglia also signifies a transition towards a more phagocytic and inflammatory phenotype (Buttgereit et al., 2016), which together implicates SHIP-1 in the regulation of microglial activation.

Acute cellular responses in the brain were also elevated post-injury, independently of SHIP-1 deficiency. Consistent with past studies in TBI (Jin et al., 2012; Loane et al., 2014; Turtzo et al., 2014; Green et al., 2022b), the activation of microglia and astrocyte (i.e., augmented IBA-1 and GFAP in the injured hemisphere) was evident at 1-week post-injury. Contrary to our initial hypothesis, we found that SHIP-1 deficiency had no effect on acute microglial activation after experimental TBI in pediatric mice. Additionally, while CD68+ macrophages were present in the injured cortex, as expected (Wang et al., 2013; Helmy et al., 2014), the extent of macrophage infiltration was not affected by SHIP-1 deficiency. This finding is in contrast to previous reports of aberrant macrophage infiltration into other organs, such as the lung parenchyma and the lamina propria of the ileum, in SHIP-1−/− mice (Maxwell et al., 2011; Park et al., 2014).

The effect of SHIP-1 deficiency on acute glial and cellular responses at this time-point may have been compensated for by other PI3K signaling pathway regulators such as Phosphatase and tensin homolog (PTEN), which attenuates glial activation following experimental chronic pain (Huang et al., 2015). Alternatively, other pathways such as ERK signaling–regulated independently of SHIP-1–can suppress acute microglial and astrocyte responses (Chen et al., 2018; Divolis et al., 2019), as well as macrophage infiltration following focal brain injury (Huang et al., 2020). The p38α-MAPK pathway may also compensate for reduced SHIP-1 inhibition, and has been shown to modulate microglial cytokine production after diffuse brain injury in rodents (Bachstetter et al., 2013). While a recent study observed no changes in ERK or MAPK signaling in microglia after *Inpp5d* knockdown in the context of neurodegenerative disease (Iguchi et al., 2023), future investigations into how SHIP-1 deficiency affects other components of the PI3K signaling pathway in microglia after brain trauma may provide further insight into these distinct or overlapping mechanisms.

Along with immune activation, pediatric TBI also resulted in behavioral deficits including increased anxiety-like behavior, reduced explorative activity, and impaired working memory, as previously reported in this model (Treble et al., 2013; Semple et al., 2014; Sharma et al., 2022). Of note, SHIP-1−/− mice exhibited reduced activity in the open-field test regardless of TBI. This genotype-dependent hypoactive phenotype was not attributed to a gross motor deficit, as all groups had comparable sensorimotor performance on the accelerating rotarod. It may, however, be a consequence of exacerbated peripheral inflammation, as previously reported in this strain (Roongapinun et al., 2010; Maxwell et al., 2011), akin to what is observed after a peripheral immune challenge in pediatric mice (Sharma et al., 2021).

The cognitive impairments and behavioral changes resulting from pediatric TBI often emerge during adolescence and adulthood (McKinlay et al., 2002; Babikian and Asarnow, 2009; Andruszkow et al., 2014; Max, 2014; Stephens et al., 2017; Hwang et al., 2019; Neumane et al., 2021), possibly as a result of neurodegeneration associated with chronic neuroinflammation (Ertürk et al., 2016; Ryan et al., 2022). As the PI3K-AKT pathway, regulated by SHIP-1, is implicated in neuroimmune responses (Cianciulli et al., 2020; Chu et al., 2021), we hypothesized that SHIP-1 deficiency would modify the immune response at a chronic time-point after pediatric TBI. At 12 weeks post-injury, both sham and injured SHIP-1−/− mice exhibited reduced body weight, pronounced splenomegaly, and elevated circulating cytokines and growth factors compared to SHIP-1+/− controls, confirming the previously characterized SHIP-1-deficient phenotype (Maeda et al., 2010; Tsantikos et al., 2018). In addition, TBI reduced the levels of IFN-γ, IL-10, and MIP-1α. While poorly characterized to date, immune suppression following acute TBI and stroke has been attributed to populations of myeloid-derived suppressor cells that antagonize pro-inflammatory responses in the periphery of TBI animals (Grundy et al., 2001; Agha et al., 2004; Urra et al., 2009; Bryk et al., 2010; Vogelgesang et al., 2010; Pillay et al., 2012; Zhang et al., 2012; Hazeldine et al., 2015; Veglia et al., 2021). Further research is required to better understand the effect of chronic TBI on systemic inflammation, and the effects of immune changes on neurological outcomes.

In the chronically-injured brain, pro-inflammatory gene expression was elevated in the absence of SHIP-1, similar to what has been observed in microglia associated with Alzheimer’s disease (Lambert et al., 2013; Farfel et al., 2016; Jing et al., 2016; Yoshino et al., 2017). While TBI did not alter cortical gene expression at 12-weeks post-injury, markers of elevated oxidative stress, neuroinflammation, blood–brain barrier breakdown, and neurodegeneration were detected in the hippocampus of TBI animals, consistent with other injury models in adult rodents (Lloyd et al., 2008; Boone et al., 2019; Hiskens et al., 2021). Of note, several inflammatory genes such as *Cd68* and *Mrc1* were further exacerbated by SHIP-1 deficiency. This finding is consistent with recent evidence suggesting that genes associated with inflammation and phagocytosis are enriched in the hippocampus of Alzheimer’s Disease models with conditional *Inpp5d* knockdown (Sierksma et al., 2020; Castranio et al., 2022). Therefore, SHIP-1 may have a distinct role in the regulation of inflammation in the hippocampus, and aberrant PI3K activity in the absence of SHIP-1 may perpetuate a pro-inflammatory response during the chronic stages of pediatric TBI.

It should be noted that exacerbated systemic inflammation in older SHIP-1−/− mice, as evident by splenomegaly and elevated circulating cytokines, may contribute to the ongoing neuroinflammation observed in the hippocampus. Inflammatory cytokines may penetrate blood brain barrier and compound neuroinflammatory responses (Qin et al., 2007; Zhao et al., 2019). Additionally, the onset of non-CNS inflammatory diseases such as ileitis, may influence microglial responses in the brain (Hanscom et al., 2021a). With increasing evidence of complex bidirectional interactions between the brain, gut, and systemic immune system (Kumar et al., 2019; Hanscom et al., 2021a,b), further investigation into the potential role of SHIP-1 in this context are warranted.

Despite the observed molecular changes, microglial activation (quantified by the amount of IBA-1 immunofluorescence, cell number, and morphology) was resolved by 12-weeks post-injury independent of SHIP-1. These results contrast with previous findings in the adult rodent brain, whereby elevated IBA-1 expression has been reported for up to 52 weeks post-TBI (Loane et al., 2014; Turtzo et al., 2014; Gazdzinski et al., 2020; Henry et al., 2020). Astrocyte reactivity was sustained at chronic timepoint consistent with previous reports (Lee et al., 2018), but surprisingly, GFAP expression around the injury site was reduced in SHIP-1-deficient mice. These glial responses in SHIP-1 deficient mice may be influenced by the presence of infiltrated regulatory T cells, given that SHIP-1 deficiency has been associated with increased proportion of circulating regulatory T cells in mice (Kashiwada et al., 2006; Collazo et al., 2009). Indeed, regulatory T cells were observed to secrete anti-inflammatory cytokines and suppresses glial responses at the cortex following CCI in mice (Krämer et al., 2019). Therefore, future investigations into how SHIP-1 deficiency may influence the adaptive immune response in the injured and diseased brain are warranted.

The ongoing neuroinflammation in the hippocampus after pediatric TBI likely promoted the extensive tissue loss seen in the ipsilateral hemisphere at the chronic time-point post-injury (Shinozaki et al., 2017). Despite elevated neuroinflammation in SHIP-1−/− mice, the volume of tissue loss remained comparable between knockout and control mice. However, the number and functionality of neurons around the injury site and hippocampus at this time-point was not examined. Further investigation into neuronal survival and activity may elucidate whether SHIP-1 plays a role in the balance between neurodegeneration and neurogenesis chronically post-TBI.

Of note, the pronounced tissue loss exhibited by TBI mice did not correspond to changes in anxiety-like behavior, general activity, motor functioning, or working memory at 12-week post-injury. However, previous studies in rodent models of TBI have shown idiosyncratic results for these outcome measures, such as differing changes in anxiety-like behavior and activity that are likely influenced by impaired GABA signaling in the injured brain (Pandey et al., 2009; Wakade et al., 2010; Schultz et al., 2011; Budinich et al., 2013; Amorós-Aguilar et al., 2015; Sierra-Mercado et al., 2015; Parga Becerra et al., 2021). In addition, motor functioning and memory has been reported to be impaired during the chronic stages of TBI; however, sex has been identified as a variable that influences performance of these tasks (Ertürk et al., 2016; Tucker et al., 2016). Several other aspects such as neuroinflammatory responses post-TBI may also be influenced by sex, as female rodents often exhibit greater anti-inflammatory responses in the brain (Moore et al., 2009; Doran et al., 2018). While historically, preclinical TBI researchers have typically used males only, we incorporated both male and female littermates in each experimental group. One limitation of the experimental design is that the number and distribution of males and females in each group were uneven due to breeding outcomes, thus not sufficient for sex to be considered statistically as a biological variable. Despite this, we did see some evidence suggestive of potential sex differences from preliminary sub-group analyzes, which warrant further investigation to elucidate the effects of sex on immune responses and behavioral changes post-TBI, in both the presence and absence of SHIP-1 activity.

Another limitation of our approach was the inability to distinguish between the intrinsic effect of SHIP-1 loss in the brain relative to the influence of peripheral inflammatory responses associated with SHIP-1 deficiency. At 12-weeks post-injury, SHIP-1−/− mice in both sham and TBI groups exhibited reduced general activity during open-field tests and memory deficits in the Y-maze. It is unclear whether these behavioral changes are a result of altered glial responses or attributed to their peripheral inflammatory cytokine infiltration into the brain (Moore et al., 2009; Bourgognon and Cavanagh, 2020). Although a global knockout model allows for the examination of the contribution of the systemic immune system to TBI responses, it is imperative for future studies to incorporate a cell-specific conditional knockout or utilization of bone marrow transplants to further understand the role of SHIP-1 and PI3K-AKT signaling in microglia *in vivo*. Cell-specific analysis of gene and protein expression changes in isolated microglia may also provide novel insight into the precise responses of these immune cells.

## Conclusion

Together, our findings suggest that SHIP-1 activity in the brain is involved in regulating chronic inflammation in the hippocampus after severe pediatric TBI, as well as potentially interacting with peripheral immune systems. However, SHIP-1 did not play a critical role in regulating microglial activation and immune responses after injury. Expression of microglial-associated inflammatory and phagocytic genes were elevated in the absence of SHIP-1, warranting further characterization through conditional SHIP-1 depletion in microglia or *ex-vivo* experiments at the steady-state brain, injured, and diseased brain. Such future studies would further delineate the role of SHIP-1 in regulating microglial responses in health and disease, with the ultimate goal of manipulating this system for potential therapeutic benefit in individuals affected by TBI.

## Data availability statement

The raw data supporting the conclusions of this article will be made available by the authors, without undue reservation.

## Ethics statement

The animal study was approved by Alfred Research Alliance Animal Ethics Committee. The study was conducted in accordance with the local legislation and institutional requirements.

## Author contributions

EC: Data curation, Formal analysis, Investigation, Methodology, Visualization, Writing – original draft, Writing – review & editing. RM: Formal analysis, Investigation, Resources, Supervision, Writing – review & editing. TG: Formal analysis, Methodology, Writing – review & editing. AZ: Data curation, Methodology, Writing – review & editing. LD: Data curation, Investigation, Methodology, Project administration, Writing – review & editing. RS: Investigation, Methodology, Writing – review & editing. AR: Investigation, Methodology, Writing – review & editing. ET: Investigation, Methodology, Resources, Writing – review & editing. MH: Conceptualization, Formal analysis, Funding acquisition, Resources, Supervision, Writing – original draft. BS: Conceptualization, Funding acquisition, Project administration, Resources, Supervision, Writing – review & editing.

## Figures and Tables

**Table 1:** 

ANOVA	Analysis of Variance
AKT	Protein Kinase B
CCI	Controlled Cortical Impact
CCL2	Chemokine (C-C motif) Ligand 2
ΔΔCT	Comparative CT Method
CNS	Central Nervous System
Crh	Corticotrophin-releasing Hormone
CSF	Colony Stimulating Factor
CX3CR1	C-X3-C Motif Chemokine Receptor 1
DAPI	4′,6’-Diamidino-2-phenlindole
GFAP	Glial Fibrillary Acidic Protein
IBA-1	Ionized Calcium-binding Adapter Molecule 1
IGFBP7	Insulin Growth Factor Binding Protein 7
IL	Interleukin
LPS	Lipopolysaccharide
Megf10	Multiple Epithelial Growth Factor (EGF) Like Domains 10
MHC II	Major Histology Complex Class II
MIP	Macrophage Inflammatory Protein
MMP	Matrix Metallopeptidase 12
NADPH	Reduced Nicotinamide Adenine Dinucleotide Phosphate
PI3K	Phosphoinositide 3-kinase
PI(3,4)P2	Phosphatidylinositol 3,4-biphosphate
PI(4,5)P2	Phosphatidylinositol 4,5-biphosphate
PI(3,4,5)P3	Phosphatidylinositol 3,4,5-triphosphate
PTEN	Phosphatase and Tensin Homolog
PCR	Real-time Quantitative Reverse Transcription Polymerase Chain Reaction
SALL1	Spalt Like Transcription Factor 1
SHIP-1	Src homology 2 domain-containing inositol polyphosphate 5-phosphatase 1
SLC1A2	Excitatory Amino Acid Transporter 2
TBI	Traumatic Brain Injury
TGF	Transforming Growth Factor
TNF	Tumor Necrosis Factor
TSPO	Translocator Protein

## References

[ref1] Agha A. Rogers B. Mylotte D. Taleb F. Tormey W. Phillips J.. (2004). Neuroendocrine dysfunction in the acute phase of traumatic brain injury. Clin. Endocrinol. 60, 584–591. doi: 10.1111/j.1365-2265.2004.02023.x, PMID: 15104561

[ref2] Amorós-Aguilar L. Portell-Cortés I. Costa-Miserachs D. Torras-Garcia M. Coll-Andreu M. (2015). Traumatic brain injury in late adolescent rats: effects on adulthood memory and anxiety. Behav. Neurosci. 129, 149–159. doi: 10.1037/bne0000046, PMID: 25730123

[ref3] Anderson C. K. Salter A. I. Toussaint L. E. Reilly E. C. Fugère C. Srivastava N.. (2015). Role of Ship1 in invariant Nkt cell development and functions. J. Immunol. 195, 2149–2156. doi: 10.4049/jimmunol.1500567, PMID: 26232432 PMC4546909

[ref4] Anderson V. Spencer-Smith M. Wood A. (2011). Do children really recover better? Neurobehavioural plasticity after early brain insult. Brain 134, 2197–2221. doi: 10.1093/brain/awr103, PMID: 21784775

[ref5] Andruszkow H. Deniz E. Urner J. Probst C. Grün O. Lohse R.. (2014). Physical and psychological long-term outcome after traumatic brain injury in children and adult patients. Health Qual. Life Outcomes 12:26. doi: 10.1186/1477-7525-12-26, PMID: 24571742 PMC3941774

[ref6] Babikian T. Asarnow R. (2009). Neurocognitive outcomes and recovery after pediatric Tbi: meta-analytic review of the literature. Neuropsychology 23, 283–296. doi: 10.1037/a0015268, PMID: 19413443 PMC4064005

[ref7] Bachstetter A. D. Rowe R. K. Kaneko M. Goulding D. Lifshitz J. Van Eldik L. J. (2013). The p38α Mapk regulates microglial responsiveness to diffuse traumatic brain injury. J. Neurosci. 33, 6143–6153. doi: 10.1523/JNEUROSCI.5399-12.2013, PMID: 23554495 PMC3667712

[ref8] Boone D. R. Weisz H. A. Willey H. E. Torres K. E. O. Falduto M. T. Sinha M.. (2019). Traumatic brain injury induces long-lasting changes in immune and regenerative signaling. PLoS One 14:e0214741. doi: 10.1371/journal.pone.0214741, PMID: 30943276 PMC6447179

[ref9] Bourgognon J. M. Cavanagh J. (2020). The role of cytokines in modulating learning and memory and brain plasticity. Brain Neurosci. Adv. 4:802. doi: 10.1177/2398212820979802, PMID: 33415308 PMC7750764

[ref10] Bryk J. A. Popovic P. J. Zenati M. S. Munera V. Pribis J. P. Ochoa J. B. (2010). Nature of myeloid cells expressing arginase 1 in peripheral blood after trauma. J. Trauma 68, 843–852. doi: 10.1097/TA.0b013e3181b026e4, PMID: 19996805

[ref11] Budinich C. S. Tucker L. B. Lowe D. Rosenberger J. G. Mccabe J. T. (2013). Short and long-term motor and behavioral effects of diazoxide and dimethyl sulfoxide administration in the mouse after traumatic brain injury. Pharmacol. Biochem. Behav. 108, 66–73. doi: 10.1016/j.pbb.2013.04.001, PMID: 23583443

[ref12] Buttgereit A. Lelios I. Yu X. Vrohlings M. Krakoski N. R. Gautier E. L.. (2016). Sall1 is a transcriptional regulator defining microglia identity and function. Nat. Immunol. 17, 1397–1406. doi: 10.1038/ni.3585, PMID: 27776109

[ref13] Cai L. Gong Q. Qi L. Xu T. Suo Q. Li X.. (2022). Act001 attenuates microglia-mediated neuroinflammation after traumatic brain injury via inhibiting Akt/NfκB/Nlrp3 pathway. Cell Commun. Signal. 20:56. doi: 10.1186/s12964-022-00862-y, PMID: 35461293 PMC9035258

[ref14] Castranio E. Hasel P. Haure-Mirande J.-V. Ramirez Jimenez A. Hamilton W. Kim R.. (2022). Inpp5D limits plaque formation and glial reactivity in the app/Ps1 mouse model of Alzheimer’s disease. bioRxiv 2022:490076. doi: 10.1101/2022.04.29.490076 PMC10481344 36448627

[ref15] Chang F. Lee J. T. Navolanic P. M. Steelman L. S. Shelton J. G. Blalock W. L.. (2003). Involvement of Pi3K/Akt pathway in cell cycle progression, apoptosis, and neoplastic transformation: a target for cancer chemotherapy. Leukemia 17, 590–603. doi: 10.1038/sj.leu.2402824, PMID: 12646949

[ref16] Chen W. Guo Y. Yang W. Chen L. Ren D. Wu C.. (2018). Phosphorylation of connexin 43 induced by traumatic brain injury promotes exosome release. J. Neurophysiol. 119, 305–311. doi: 10.1152/jn.00654.2017, PMID: 29046426

[ref17] Chu E. Mychasiuk R. Hibbs M. L. Semple B. D. (2021). Dysregulated phosphoinositide 3-kinase signaling in microglia: shaping chronic neuroinflammation. J. Neuroinflammation 18:276. doi: 10.1186/s12974-021-02325-6, PMID: 34838047 PMC8627624

[ref18] Cianciulli A. Porro C. Calvello R. Trotta T. Lofrumento D. D. Panaro M. A. (2020). Microglia mediated Neuroinflammation: focus on Pi3K modulation. Biomol. Ther. 10:137. doi: 10.3390/biom10010137, PMID: 31947676 PMC7022557

[ref19] Collazo M. M. Wood D. Paraiso K. H. Lund E. Engelman R. W. Le C. T.. (2009). Ship limits immunoregulatory capacity in the T-cell compartment. Blood 113, 2934–2944. doi: 10.1182/blood-2008-09-181164, PMID: 19136659 PMC2662640

[ref20] Desponts C. Hazen A. L. Paraiso K. H. Kerr W. G. (2006). Ship deficiency enhances Hsc proliferation and survival but compromises homing and repopulation. Blood 107, 4338–4345. doi: 10.1182/blood-2005-12-5021, PMID: 16467196 PMC1464834

[ref21] Dewan M. C. Mummareddy N. Wellons J. C. Bonfield C. M. (2016). Epidemiology of global pediatric traumatic brain injury: qualitative review. World Neurosurg. 91, 497–509.e1. doi: 10.1016/j.wneu.2016.03.045, PMID: 27018009

[ref22] Divolis G. Stavropoulos A. Manioudaki M. Apostolidou A. Doulou A. Gavriil A.. (2019). Activation of both transforming growth factor-β and bone morphogenetic protein signalling pathways upon traumatic brain injury restrains pro-inflammatory and boosts tissue reparatory responses of reactive astrocytes and microglia. Brain Commun. 1:fcz028. doi: 10.1093/braincomms/fcz028, PMID: 32954268 PMC7425383

[ref23] Donat C. K. Scott G. Gentleman S. M. Sastre M. (2017). Microglial activation in traumatic brain injury. Front. Aging Neurosci. 9:208. doi: 10.3389/fnagi.2017.00208, PMID: 28701948 PMC5487478

[ref24] Doran S. J. Ritzel R. M. Glaser E. P. Henry R. J. Faden A. I. Loane D. J. (2018). Sex differences in acute Neuroinflammation after experimental traumatic brain injury are mediated by infiltrating myeloid cells. J. Neurotrauma 36, 1040–1053. doi: 10.1089/neu.2018.6019, PMID: 30259790 PMC6444913

[ref25] Efthymiou A. G. Goate A. M. (2017). Late onset Alzheimer's disease genetics implicates microglial pathways in disease risk. Mol. Neurodegener. 12:43. doi: 10.1186/s13024-017-0184-x, PMID: 28549481 PMC5446752

[ref26] Ertürk A. Mentz S. Stout E. E. Hedehus M. Dominguez S. L. Neumaier L.. (2016). Interfering with the chronic immune response rescues chronic degeneration after traumatic brain injury. J. Neurosci. 36, 9962–9975. doi: 10.1523/JNEUROSCI.1898-15.2016, PMID: 27656033 PMC6705567

[ref27] Farfel J. M. Yu L. Buchman A. S. Schneider J. A. De Jager P. L. Bennett D. A. (2016). Relation of genomic variants for Alzheimer disease dementia to common neuropathologies. Neurology 87, 489–496. doi: 10.1212/WNL.0000000000002909, PMID: 27371493 PMC4970661

[ref28] Gage G. J. Kipke D. R. Shain W. (2012). Whole animal perfusion fixation for rodents. J. Vis. Exp. 65:3564. doi: 10.3791/3564, PMID: 22871843 PMC3476408

[ref29] Gazdzinski L. M. Mellerup M. Wang T. Adel S. A. A. Lerch J. P. Sled J. G.. (2020). White matter changes caused by mild traumatic brain injury in mice evaluated using neurite orientation dispersion and density imaging. J. Neurotrauma 37, 1818–1828. doi: 10.1089/neu.2020.6992, PMID: 32242488

[ref30] Girgis F. Pace J. Sweet J. Miller J. P. (2016). Hippocampal neurophysiologic changes after mild traumatic brain injury and potential neuromodulation treatment approaches. Front. Syst. Neurosci. 10:8. doi: 10.3389/fnsys.2016.00008, PMID: 26903824 PMC4746250

[ref31] Green T. R. F. Murphy S. M. Moreno-Montano M. P. Audinat E. Rowe R. K. (2022a). Reactive morphology of dividing microglia following kainic acid administration. Front. Neurosci. 16:972138. doi: 10.3389/fnins.2022.972138, PMID: 36248637 PMC9556904

[ref32] Green T. R. F. Murphy S. M. Ortiz J. B. Rowe R. K. (2022b). Age-at-injury influences the glial response to traumatic brain injury in the cortex of male juvenile rats. Front. Neurol. 12:804139. doi: 10.3389/fneur.2021.804139, PMID: 35111130 PMC8802670

[ref33] Green T. R. F. Murphy S. M. Rowe R. K. (2022c). Comparisons of quantitative approaches for assessing microglial morphology reveal inconsistencies, ecological fallacy, and a need for standardization. Sci. Rep. 12:18196. doi: 10.1038/s41598-022-23091-2, PMID: 36307475 PMC9616881

[ref34] Grundy P. L. Harbuz M. S. Jessop D. S. Lightman S. L. Sharples P. M. (2001). The hypothalamo-pituitary-adrenal axis response to experimental traumatic brain injury. J. Neurotrauma 18, 1373–1381. doi: 10.1089/08977150152725669, PMID: 11780867

[ref35] Haddon D. Antignano F. Hughes M. Blanchet M.-R. Zbytnuik L. Krystal G.. (2009). Ship1 is a repressor of mast cell hyperplasia, cytokine production, and allergic inflammation in vivo. J. Immunol. 183, 228–236. doi: 10.4049/jimmunol.0900427, PMID: 19542434

[ref36] Hanscom M. Loane D. J. Aubretch T. Leser J. Molesworth K. Hedgekar N.. (2021a). Acute colitis during chronic experimental traumatic brain injury in mice induces dysautonomia and persistent extraintestinal, systemic, and Cns inflammation with exacerbated neurological deficits. J. Neuroinflammation 18:24. doi: 10.1186/s12974-020-02067-x, PMID: 33461596 PMC7814749

[ref37] Hanscom M. Loane D. J. Shea-Donohue T. (2021b). Brain-gut axis dysfunction in the pathogenesis of traumatic brain injury. J. Clin. Invest. 131:e143777. doi: 10.1172/JCI143777, PMID: 34128471 PMC8203445

[ref38] Hazeldine J. Lord J. M. Belli A. (2015). Traumatic brain injury and peripheral immune suppression: primer and prospectus. Front. Neurol. 6:235. doi: 10.3389/fneur.2015.00235, PMID: 26594196 PMC4633482

[ref39] Helgason C. D. Damen J. E. Rosten P. Grewal R. Sorensen P. Chappel S. M.. (1998). Targeted disruption of Ship leads to hemopoietic perturbations, lung pathology, and a shortened life span. Genes Dev. 12, 1610–1620. doi: 10.1101/gad.12.11.1610, PMID: 9620849 PMC316868

[ref40] Helmy A. Guilfoyle M. R. Carpenter K. L. Pickard J. D. Menon D. K. Hutchinson P. J. (2014). Recombinant human interleukin-1 receptor antagonist in severe traumatic brain injury: a phase ii randomized control trial. J. Cereb. Blood Flow Metab. 34, 845–851. doi: 10.1038/jcbfm.2014.23, PMID: 24569690 PMC4013762

[ref41] Henry R. J. Ritzel R. M. Barrett J. P. Doran S. J. Jiao Y. Leach J. B.. (2020). Microglial depletion with Csf1R inhibitor during chronic phase of experimental traumatic brain injury reduces neurodegeneration and neurological deficits. J. Neurosci. 40, 2960–2974. doi: 10.1523/JNEUROSCI.2402-19.2020, PMID: 32094203 PMC7117897

[ref42] Hibbs M. L. Raftery A. L. Tsantikos E. (2018). Regulation of hematopoietic cell signaling by Ship-1 inositol phosphatase: growth factors and beyond. Growth Factors 36, 213–231. doi: 10.1080/08977194.2019.1569649, PMID: 30764683

[ref43] Hickman S. E. Kingery N. D. Ohsumi T. K. Borowsky M. L. Wang L. C. Means T. K.. (2013). The microglial sensome revealed by direct Rna sequencing. Nat. Neurosci. 16, 1896–1905. doi: 10.1038/nn.3554, PMID: 24162652 PMC3840123

[ref44] Hiskens M. I. Schneiders A. G. Vella R. K. Fenning A. S. (2021). Repetitive mild traumatic brain injury affects inflammation and excitotoxic mrna expression at acute and chronic time-points. PLoS One 16:e0251315. doi: 10.1371/journal.pone.0251315, PMID: 33961674 PMC8104440

[ref45] Huang Y. Li Q. Tian H. Yao X. Bakina O. Zhang H.. (2020). Mek inhibitor trametinib attenuates neuroinflammation and cognitive deficits following traumatic brain injury in mice. Am. J. Transl. Res. 12, 6351–6365. PMID: 33194035 PMC7653601

[ref46] Huang S. Y. Sung C. S. Chen W. F. Chen C. H. Feng C. W. Yang S. N.. (2015). Involvement of phosphatase and tensin homolog deleted from chromosome 10 in rodent model of neuropathic pain. J. Neuroinflammation 12:59. doi: 10.1186/s12974-015-0280-1, PMID: 25889774 PMC4386079

[ref47] Huang X. You W. Zhu Y. Xu K. Yang X. Wen L. (2021). Microglia: a potential drug target for traumatic axonal injury. Neural Plast. 2021:5554824. doi: 10.1155/2021/5554824, PMID: 34093701 PMC8163545

[ref48] Hwang S. Y. Ong J. W. Ng Z. M. Foo C. Y. Chua S. Z. Sri D.. (2019). Long-term outcomes in children with moderate to severe traumatic brain injury: a single-Centre retrospective study. Brain Inj. 33, 1420–1424. doi: 10.1080/02699052.2019.1641625, PMID: 31314599

[ref49] Iguchi A. Takatori S. Kimura S. Muneto H. Wang K. Etani H.. (2023). Inpp5D modulates Trem2 loss-of-function phenotypes in a β-amyloidosis mouse model. iScience 26:106375. doi: 10.1016/j.isci.2023.106375, PMID: 37035000 PMC10074152

[ref50] Israelsson C. Wang Y. Kylberg A. Pick C. G. Hoffer B. J. Ebendal T. (2009). Closed head injury in a mouse model results in molecular changes indicating inflammatory responses. J. Neurotrauma 26, 1307–1314. doi: 10.1089/neu.2008.0676, PMID: 19317611 PMC2989856

[ref51] Jin X. Ishii H. Bai Z. Itokazu T. Yamashita T. (2012). Temporal changes in cell marker expression and cellular infiltration in a controlled cortical impact model in adult male C57bl/6 mice. PLoS One 7:e41892. doi: 10.1371/journal.pone.0041892, PMID: 22911864 PMC3404031

[ref52] Jing H. Zhu J.-X. Wang H.-F. Zhang W. Zheng Z.-J. Kong L.-L.. (2016). Inpp5D rs35349669 polymorphism with late-onset Alzheimer's disease: a replication study and meta-analysis. Oncotarget 7, 69225–69230. doi: 10.18632/oncotarget.12648, PMID: 27750211 PMC5342472

[ref53] Johnson V. E. Stewart J. E. Begbie F. D. Trojanowski J. Q. Smith D. H. Stewart W. (2013). Inflammation and white matter degeneration persist for years after a single traumatic brain injury. Brain 136, 28–42. doi: 10.1093/brain/aws322, PMID: 23365092 PMC3562078

[ref54] Kamen L. A. Levinsohn J. Cadwallader A. Tridandapani S. Swanson J. A. (2008). Ship-1 increases early oxidative burst and regulates phagosome maturation in macrophages. J. Immunol. 180, 7497–7505. doi: 10.4049/jimmunol.180.11.7497, PMID: 18490750 PMC2913413

[ref55] Kashiwada M. Cattoretti G. Mckeag L. Rouse T. Showalter B. M. Al-Alem U.. (2006). Downstream of tyrosine kinases-1 and Src homology 2-containing inositol 5′-phosphatase are required for regulation of Cd4+Cd25+ T cell development. J. Immunol. 176, 3958–3965. doi: 10.4049/jimmunol.176.7.3958, PMID: 16547230

[ref56] Kerr W. G. Park M.-Y. Maubert M. Engelman R. W. (2011). Ship deficiency causes Crohn's disease-like ileitis. Gut 60, 177–188. doi: 10.1136/gut.2009.202283, PMID: 20940287 PMC3022365

[ref57] Kigerl K. A. Gensel J. C. Ankeny D. P. Alexander J. K. Donnelly D. J. Popovich P. G. (2009). Identification of two distinct macrophage subsets with divergent effects causing either neurotoxicity or regeneration in the injured mouse spinal cord. J. Neurosci. 29, 13435–13444. doi: 10.1523/JNEUROSCI.3257-09.2009, PMID: 19864556 PMC2788152

[ref58] Kinoshita K. (2016). Traumatic brain injury: pathophysiology for neurocritical care. J. Intensive Care 4:29. doi: 10.1186/s40560-016-0138-3, PMID: 27123305 PMC4847183

[ref59] Kolb B. Cioe J. Whishaw I. Q. (2000). Is there an optimal age for recovery from motor cortex lesions? I. behavioral and anatomical sequelae of bilateral motor cortex lesions in rats on postnatal days 1, 10, and in adulthood. Brain Res. 882, 62–74. doi: 10.1016/S0006-8993(00)02828-6, PMID: 11056185

[ref60] Kostandy B. B. (2012). The role of glutamate in neuronal ischemic injury: the role of spark in fire. Neurol. Sci. 33, 223–237. doi: 10.1007/s10072-011-0828-5, PMID: 22044990

[ref61] Koyasu S. (2003). The role of Pi3K in immune cells. Nat. Immunol. 4, 313–319. doi: 10.1038/ni0403-313, PMID: 12660731

[ref62] Krämer T. J. Hack N. Brühl T. J. Menzel L. Hummel R. Griemert E. V.. (2019). Correction to: depletion of regulatory T cells increases T cell brain infiltration, reactive astrogliosis, and interferon-γ gene expression in acute experimental traumatic brain injury. J. Neuroinflammation 16:176. doi: 10.1186/s12974-019-1577-2, PMID: 31493788 PMC6731564

[ref63] Kumar A. Carrillo L. A. Cardenas F. B. Toledano-Furman N. Cox C. S. (2019). Gut Dysbiosis leads to increased microglial activation and inflammatory response acutely after traumatic brain injury in rats. J. Am. Coll. Surg. 229:S296. doi: 10.1016/j.jamcollsurg.2019.08.648

[ref64] Lambert J. C. Ibrahim-Verbaas C. A. Harold D. Naj A. C. Sims R. Bellenguez C.. (2013). Meta-analysis of 74,046 individuals identifies 11 new susceptibility loci for Alzheimer's disease. Nat. Genet. 45, 1452–1458. doi: 10.1038/ng.2802, PMID: 24162737 PMC3896259

[ref65] Lee S. W. Gajavelli S. Spurlock M. S. Andreoni C. De Rivero Vaccari J. P. Bullock M. R.. (2018). Microglial Inflammasome activation in penetrating ballistic-like brain injury. J. Neurotrauma 35, 1681–1693. doi: 10.1089/neu.2017.5530, PMID: 29439605 PMC6016174

[ref66] Lenzlinger P. M. Hans V. H. Jöller-Jemelka H. I. Trentz O. Morganti-Kossmann M. C. Kossmann T. (2001). Markers for cell-mediated immune response are elevated in cerebrospinal fluid and serum after severe traumatic brain injury in humans. J. Neurotrauma 18, 479–489. doi: 10.1089/089771501300227288, PMID: 11393251

[ref67] Lin P. B.-C. Tsai A. P.-Y. Nho K. Lamb B. T. Oblak A. L. (2021). Inpp5D as a potential therapeutic target against Alzheimer’s disease. Alzheimers Dement. 17:e053236. doi: 10.1002/alz.053236

[ref68] Lindblad C. Pin E. Just D. Al Nimer F. Nilsson P. Bellander B. M.. (2021). Fluid proteomics of Csf and serum reveal important neuroinflammatory proteins in blood-brain barrier disruption and outcome prediction following severe traumatic brain injury: a prospective, observational study. Crit. Care 25:103. doi: 10.1186/s13054-021-03503-x, PMID: 33712077 PMC7955664

[ref69] Liu Q. Sasaki T. Kozieradzki I. Wakeham A. Itie A. Dumont D. J.. (1999). Ship is a negative regulator of growth factor receptor-mediated Pkb/Akt activation and myeloid cell survival. Genes Dev. 13, 786–791. doi: 10.1101/gad.13.7.786, PMID: 10197978 PMC316591

[ref70] Lloyd E. Somera-Molina K. Van Eldik L. J. Watterson D. M. Wainwright M. S. (2008). Suppression of acute proinflammatory cytokine and chemokine upregulation by post-injury administration of a novel small molecule improves long-term neurologic outcome in a mouse model of traumatic brain injury. J. Neuroinflammation 5:28. doi: 10.1186/1742-2094-5-28, PMID: 18590543 PMC2483713

[ref71] Loane D. J. Kumar A. Stoica B. A. Cabatbat R. Faden A. I. (2014). Progressive neurodegeneration after experimental brain trauma: association with chronic microglial activation. J. Neuropathol. Exp. Neurol. 73, 14–29. doi: 10.1097/NEN.0000000000000021, PMID: 24335533 PMC4267248

[ref72] Maeda K. Mehta H. Drevets D. A. Coggeshall K. M. (2010). Il-6 increases B-cell IgG production in a feed-forward proinflammatory mechanism to skew hematopoiesis and elevate myeloid production. Blood 115, 4699–4706. doi: 10.1182/blood-2009-07-230631, PMID: 20351305 PMC3790945

[ref73] Mahajan S. S. Thai K. H. Chen K. Ziff E. (2011). Exposure of neurons to excitotoxic levels of glutamate induces cleavage of the Rna editing enzyme, adenosine deaminase acting on Rna 2, and loss of Glur2 editing. Neuroscience 189, 305–315. doi: 10.1016/j.neuroscience.2011.05.027, PMID: 21620933 PMC3150305

[ref74] Marcos R. Monteiro R. A. F. Rocha E. (2012). The use of design-based stereology to evaluate volumes and numbers in the liver: a review with practical guidelines. J. Anat. 220, 303–317. doi: 10.1111/j.1469-7580.2012.01475.x, PMID: 22296163 PMC3375768

[ref75] Marsh S. E. Walker A. J. Kamath T. Dissing-Olesen L. Hammond T. R. De Soysa T. Y.. (2022). Dissection of artifactual and confounding glial signatures by single-cell sequencing of mouse and human brain. Nat. Neurosci. 25, 306–316. doi: 10.1038/s41593-022-01022-8, PMID: 35260865 PMC11645269

[ref76] Marzolo M. P. Von Bernhardi R. Inestrosa N. C. (1999). Mannose receptor is present in a functional state in rat microglial cells. J. Neurosci. Res. 58, 387–395. doi: 10.1002/(SICI)1097-4547(19991101)58:3<387::AID-JNR4>3.0.CO;2-L, PMID: 10518112

[ref77] Max J. E. (2014). Neuropsychiatry of pediatric traumatic brain injury. Psychiatr. Clin. North Am. 37, 125–140. doi: 10.1016/j.psc.2013.11.003, PMID: 24529428 PMC3977029

[ref78] Maxwell M. J. Duan M. Armes J. E. Anderson G. P. Tarlinton D. M. Hibbs M. L. (2011). Genetic segregation of inflammatory lung disease and autoimmune disease severity in Ship-1−/− mice. J. Immunol. 186, 7164–7175. doi: 10.4049/jimmunol.1004185, PMID: 21572033

[ref79] Mckinlay A. Dalrymple-Alford J. C. Horwood L. J. Fergusson D. M. (2002). Long term psychosocial outcomes after mild head injury in early childhood. J. Neurol. Neurosurg. Psychiatry 73, 281–288. doi: 10.1136/jnnp.73.3.281, PMID: 12185159 PMC1738032

[ref80] Mclarren K. W. Cole A. E. Weisser S. B. Voglmaier N. S. Conlin V. S. Jacobson K.. (2011). Ship-deficient mice develop spontaneous intestinal inflammation and arginase-dependent fibrosis. Am. J. Pathol. 179, 180–188. doi: 10.1016/j.ajpath.2011.03.018, PMID: 21640975 PMC3123870

[ref81] Moore A. H. Wu M. Shaftel S. S. Graham K. A. O'banion M. K. (2009). Sustained expression of interleukin-1beta in mouse hippocampus impairs spatial memory. Neuroscience 164, 1484–1495. doi: 10.1016/j.neuroscience.2009.08.073, PMID: 19744544 PMC2783232

[ref82] Morrison H. Young K. Qureshi M. Rowe R. K. Lifshitz J. (2017). Quantitative microglia analyses reveal diverse morphologic responses in the rat cortex after diffuse brain injury. Sci. Rep. 7:13211. doi: 10.1038/s41598-017-13581-z, PMID: 29038483 PMC5643511

[ref83] Neumane S. Câmara-Costa H. Francillette L. Araujo M. Toure H. Brugel D.. (2021). Functional outcome after severe childhood traumatic brain injury: results of the Tge prospective longitudinal study. Ann. Phys. Rehabil. Med. 64:101375. doi: 10.1016/j.rehab.2020.01.008, PMID: 32275965

[ref84] Olah M. Patrick E. Villani A.-C. Xu J. White C. C. Ryan K. J.. (2018). A transcriptomic atlas of aged human microglia. Nat. Commun. 9:539. doi: 10.1038/s41467-018-02926-5, PMID: 29416036 PMC5803269

[ref85] Pandey D. K. Yadav S. K. Mahesh R. Rajkumar R. (2009). Depression-like and anxiety-like behavioural aftermaths of impact accelerated traumatic brain injury in rats: a model of comorbid depression and anxiety? Behav. Brain Res. 205, 436–442. doi: 10.1016/j.bbr.2009.07.027, PMID: 19660499

[ref86] Parga Becerra A. Logsdon A. F. Banks W. A. Ransom C. B. (2021). Traumatic brain injury broadly affects Gabaergic signaling in dentate gyrus granule cells. eNeuro 8:55. doi: 10.1523/ENEURO.0055-20.2021, PMID: 33514602 PMC8116114

[ref87] Park M. Y. Srivastava N. Sudan R. Viernes D. R. Chisholm J. D. Engelman R. W.. (2014). Impaired T-cell survival promotes mucosal inflammatory disease in Ship1-deficient mice. Mucosal Immunol. 7, 1429–1439. doi: 10.1038/mi.2014.32, PMID: 24781051 PMC4205272

[ref88] Pedicone C. Fernandes S. Dungan O. M. Dormann S. M. Viernes D. R. Adhikari A. A.. (2020). Pan-Ship1/2 inhibitors promote microglia effector functions essential for Cns homeostasis. J. Cell Sci. 133:jcs238030. doi: 10.1242/jcs.238030, PMID: 31780579 PMC10682645

[ref89] Pillay J. Kamp V. M. Van Hoffen E. Visser T. Tak T. Lammers J. W.. (2012). A subset of neutrophils in human systemic inflammation inhibits T cell responses through mac-1. J. Clin. Invest. 122, 327–336. doi: 10.1172/JCI57990, PMID: 22156198 PMC3248287

[ref90] Qin L. Wu X. Block M. L. Liu Y. Breese G. R. Hong J. S.. (2007). Systemic Lps causes chronic neuroinflammation and progressive neurodegeneration. Glia 55, 453–462. doi: 10.1002/glia.20467, PMID: 17203472 PMC2871685

[ref91] Raghavendra Rao V. L. Dogan A. Bowen K. K. Dempsey R. J. (2000). Traumatic brain injury leads to increased expression of peripheral-type benzodiazepine receptors, neuronal death, and activation of astrocytes and microglia in rat thalamus. Exp. Neurol. 161, 102–114. doi: 10.1006/exnr.1999.7269, PMID: 10683277

[ref92] Raghupathi R. Huh J. W. (2017). Age-at-injury effects of microglial activation following traumatic brain injury: implications for treatment strategies. Neural Regen. Res. 12, 741–742. doi: 10.4103/1673-5374.206639, PMID: 28616025 PMC5461606

[ref93] Rice R. A. Spangenberg E. E. Yamate-Morgan H. Lee R. J. Arora R. P. S. Hernandez M. X.. (2015). Elimination of microglia improves functional outcomes following extensive neuronal loss in the Hippocampus. J. Neurosci. 35, 9977–9989. doi: 10.1523/JNEUROSCI.0336-15.2015, PMID: 26156998 PMC4495246

[ref94] Roongapinun S. Oh S.-Y. Wu F. Panthong A. Zheng T. Zhu Z. (2010). Role of Ship-1 in the adaptive immune responses to aeroallergen in the airway. PLoS One 5:e14174. doi: 10.1371/journal.pone.0014174, PMID: 21151496 PMC2994819

[ref95] Ryan E. Kelly L. Stacey C. Huggard D. Duff E. Mccollum D.. (2022). Mild-to-severe traumatic brain injury in children: altered cytokines reflect severity. J. Neuroinflammation 19:36. doi: 10.1186/s12974-022-02390-5, PMID: 35130911 PMC8822689

[ref96] Ryu J. Stone P. Lee S. Payne B. Gorse K. Lafrenaye A. (2021). Buprenorphine alters microglia and astrocytes acutely following diffuse traumatic brain injury. Sci. Rep. 11:8620. doi: 10.1038/s41598-021-88030-z, PMID: 33883663 PMC8060410

[ref97] Scheid M. P. Woodgett J. R. (2001). Pkb/Akt: functional insights from genetic models. Nat. Rev. Mol. Cell Biol. 2, 760–768. doi: 10.1038/35096067, PMID: 11584303

[ref98] Schultz B. A. Cifu D. X. Mcnamee S. Nichols M. Carne W. (2011). Assessment and treatment of common persistent sequelae following blast induced mild traumatic brain injury. NeuroRehabilitation 28, 309–320. doi: 10.3233/NRE-2011-0659, PMID: 21725164

[ref99] Semple B. D. Noble-Haeusslein L. J. Jun Kwon Y. Sam P. N. Gibson A. M. Grissom S.. (2014). Sociosexual and communication deficits after traumatic injury to the developing murine brain. PLoS One 9:e103386. doi: 10.1371/journal.pone.0103386, PMID: 25106033 PMC4126664

[ref100] Semple B. D. O'brien T. J. Gimlin K. Wright D. K. Kim S. E. Casillas-Espinosa P. M.. (2017). Interleukin-1 receptor in seizure susceptibility after traumatic injury to the pediatric brain. J. Neurosci. 37, 7864–7877. doi: 10.1523/JNEUROSCI.0982-17.2017, PMID: 28724747 PMC5559762

[ref101] Sharma R. Casillas-Espinosa P. M. Dill L. K. Rewell S. S. J. Hudson M. R. O'brien T. J.. (2022). Pediatric traumatic brain injury and a subsequent transient immune challenge independently influenced chronic outcomes in male mice. Brain Behav. Immun. 100, 29–47. doi: 10.1016/j.bbi.2021.11.013, PMID: 34808288

[ref102] Sharma R. Chu E. Dill L. K. Shad A. Zamani A. O'brien T. J.. (2023). Ccr2 gene ablation does not influence seizure susceptibility, tissue damage, or cellular inflammation after murine pediatric traumatic brain injury. J. Neurotrauma 40, 365–382. doi: 10.1089/neu.2022.0033, PMID: 36070444

[ref103] Sharma R. Zamani A. Dill L. K. Sun M. Chu E. Robinson M. J.. (2021). A systemic immune challenge to model hospital-acquired infections independently regulates immune responses after pediatric traumatic brain injury. J. Neuroinflammation 18:72. doi: 10.1186/s12974-021-02114-1, PMID: 33731173 PMC7968166

[ref104] Shinozaki Y. Shibata K. Yoshida K. Shigetomi E. Gachet C. Ikenaka K.. (2017). Transformation of astrocytes to a neuroprotective phenotype by microglia via P2Y1 receptor downregulation. Cell Rep. 19, 1151–1164. doi: 10.1016/j.celrep.2017.04.047, PMID: 28494865

[ref105] Shultz S. R. Shah A. D. Huang C. Dill L. K. Schittenhelm R. B. Morganti-Kossmann M. C.. (2022). Temporal proteomics of human cerebrospinal fluid after severe traumatic brain injury. J. Neuroinflammation 19:291. doi: 10.1186/s12974-022-02654-0, PMID: 36482407 PMC9730674

[ref106] Sierksma A. Lu A. Mancuso R. Fattorelli N. Thrupp N. Salta E.. (2020). Novel Alzheimer risk genes determine the microglia response to amyloid-β but not to tau pathology. EMBO Mol. Med. 12:e10606. doi: 10.15252/emmm.201910606, PMID: 31951107 PMC7059012

[ref107] Sierra-Mercado D. Mcallister L. M. Lee C. C. Milad M. R. Eskandar E. N. Whalen M. J. (2015). Controlled cortical impact before or after fear conditioning does not affect fear extinction in mice. Brain Res. 1606, 133–141. doi: 10.1016/j.brainres.2015.02.031, PMID: 25721797 PMC4518729

[ref108] Stephens J. Salorio C. Denckla M. Mostofsky S. Suskauer S. (2017). Subtle motor findings during recovery from pediatric traumatic brain injury: a preliminary report. J. Mot. Behav. 49, 20–26. doi: 10.1080/00222895.2016.1204267, PMID: 27635631 PMC5356925

[ref109] Takeshita S. Namba N. Zhao J. J. Jiang Y. Genant H. K. Silva M. J.. (2002). Ship-deficient mice are severely osteoporotic due to increased numbers of hyper-resorptive osteoclasts. Nat. Med. 8, 943–949. doi: 10.1038/nm752, PMID: 12161749

[ref110] Tasker R. C. Salmond C. H. Westland A. G. Pena A. Gillard J. H. Sahakian B. J.. (2005). Head circumference and brain and hippocampal volume after severe traumatic brain injury in childhood. Pediatr. Res. 58, 302–308. doi: 10.1203/01.PDR.0000169965.08854.25, PMID: 16006434

[ref111] Tong W. Igarashi T. Ferriero D. M. Noble L. J. (2002). Traumatic brain injury in the immature mouse brain: characterization of regional vulnerability. Exp. Neurol. 176, 105–116. doi: 10.1006/exnr.2002.7941, PMID: 12093087

[ref112] Treble A. Hasan K. M. Iftikhar A. Stuebing K. K. Kramer L. A. Cox C. S. Jr.. (2013). Working memory and corpus callosum microstructural integrity after pediatric traumatic brain injury: a diffusion tensor tractography study. J. Neurotrauma 30, 1609–1619. doi: 10.1089/neu.2013.2934, PMID: 23627735 PMC3787334

[ref113] Tsai A. P. Lin P. B.-C. Dong C. Moutinho M. Casali B. T. Liu Y.. (2021). Inpp5D expression is associated with risk for Alzheimer's disease and induced by plaque-associated microglia. Neurobiol. Dis. 153:105303. doi: 10.1016/j.nbd.2021.105303, PMID: 33631273 PMC8082515

[ref114] Tsantikos E. Lau M. Castelino C. M. Maxwell M. J. Passey S. L. Hansen M. J.. (2018). Granulocyte-Csf links destructive inflammation and comorbidities in obstructive lung disease. J. Clin. Invest. 128, 2406–2418. doi: 10.1172/JCI98224, PMID: 29708507 PMC5983324

[ref115] Tucker L. B. Fu A. H. Mccabe J. T. (2016). Performance of male and female C57bl/6J mice on motor and cognitive tasks commonly used in pre-clinical traumatic brain injury research. J. Neurotrauma 33, 880–894. doi: 10.1089/neu.2015.3977, PMID: 25951234 PMC4860656

[ref116] Turtzo L. C. Lescher J. Janes L. Dean D. D. Budde M. D. Frank J. A. (2014). Macrophagic and microglial responses after focal traumatic brain injury in the female rat. J. Neuroinflammation 11:82. doi: 10.1186/1742-2094-11-82, PMID: 24761998 PMC4022366

[ref117] Urra X. Cervera A. Obach V. Climent N. Planas A. M. Chamorro A. (2009). Monocytes are major players in the prognosis and risk of infection after acute stroke. Stroke 40, 1262–1268. doi: 10.1161/STROKEAHA.108.532085, PMID: 19164783

[ref118] Veglia F. Sanseviero E. Gabrilovich D. I. (2021). Myeloid-derived suppressor cells in the era of increasing myeloid cell diversity. Nat. Rev. Immunol. 21, 485–498. doi: 10.1038/s41577-020-00490-y, PMID: 33526920 PMC7849958

[ref119] Viviani B. Boraso M. Valero M. Gardoni F. Marco E. M. Llorente R.. (2014). Early maternal deprivation immunologically primes hippocampal synapses by redistributing interleukin-1 receptor type I in a sex dependent manner. Brain Behav. Immun. 35, 135–143. doi: 10.1016/j.bbi.2013.09.008, PMID: 24060584

[ref120] Vogelgesang A. May V. E. Grunwald U. Bakkeboe M. Langner S. Wallaschofski H.. (2010). Functional status of peripheral blood T-cells in ischemic stroke patients. PLoS One 5:e8718. doi: 10.1371/journal.pone.0008718, PMID: 20090932 PMC2806837

[ref121] Wakade C. Sukumari-Ramesh S. Laird M. D. Dhandapani K. M. Vender J. R. (2010). Delayed reduction in hippocampal postsynaptic density protein-95 expression temporally correlates with cognitive dysfunction following controlled cortical impact in mice. J. Neurosurg. 113, 1195–1201. doi: 10.3171/2010.3.JNS091212, PMID: 20397893 PMC3155981

[ref122] Wang G. Zhang J. Hu X. Zhang L. Mao L. Jiang X.. (2013). Microglia/macrophage polarization dynamics in white matter after traumatic brain injury. J. Cereb. Blood Flow Metab. 33, 1864–1874. doi: 10.1038/jcbfm.2013.146, PMID: 23942366 PMC3851898

[ref123] Webster K. M. Shultz S. R. Ozturk E. Dill L. K. Sun M. Casillas-Espinosa P.. (2019a). Targeting high-mobility group box protein 1 (Hmgb1) in pediatric traumatic brain injury: chronic neuroinflammatory, behavioral, and epileptogenic consequences. Exp. Neurol. 320:112979. doi: 10.1016/j.expneurol.2019.112979, PMID: 31229637

[ref124] Webster K. M. Sun M. Crack P. J. O'brien T. J. Shultz S. R. Semple B. D. (2019b). Age-dependent release of high-mobility group box protein-1 and cellular neuroinflammation after traumatic brain injury in mice. J. Comp. Neurol. 527, 1102–1117. doi: 10.1002/cne.24589, PMID: 30499129

[ref125] Wilde E. A. Bigler E. D. Hunter J. V. Fearing M. A. Scheibel R. S. Newsome M. R.. (2007). Hippocampus, amygdala, and basal ganglia morphometrics in children after moderate-to-severe traumatic brain injury. Dev. Med. Child Neurol. 49, 294–299. doi: 10.1111/j.1469-8749.2007.00294.x, PMID: 17376141

[ref126] Witcher K. G. Bray C. E. Chunchai T. Zhao F. O'neil S. M. Gordillo A. J.. (2021). Traumatic brain injury causes chronic cortical inflammation and neuronal dysfunction mediated by microglia. J. Neurosci. 41, 1597–1616. doi: 10.1523/JNEUROSCI.2469-20.2020, PMID: 33452227 PMC7896020

[ref127] Woodcock T. Morganti-Kossmann C. (2013). The role of markers of inflammation in traumatic brain injury. Front. Neurol. 4:18. doi: 10.3389/fneur.2013.00018, PMID: 23459929 PMC3586682

[ref128] Xiao L. Gong L. L. Yuan D. Deng M. Zeng X. M. Chen L. L.. (2010). Protein phosphatase-1 regulates Akt1 signal transduction pathway to control gene expression, cell survival and differentiation. Cell Death Differ. 17, 1448–1462. doi: 10.1038/cdd.2010.16, PMID: 20186153

[ref129] Xie S. Chen M. Yan B. He X. Chen X. Li D. (2014). Identification of a role for the Pi3K/Akt/mtor signaling pathway in innate immune cells. PLoS One 9:e94496. doi: 10.1371/journal.pone.0094496, PMID: 24718556 PMC3981814

[ref130] Yoshino Y. Yamazaki K. Ozaki Y. Sao T. Yoshida T. Mori T.. (2017). Inpp5D mrna expression and cognitive decline in Japanese Alzheimer's disease subjects. J. Alzheimers Dis. 58, 687–694. doi: 10.3233/JAD-161211, PMID: 28482637

[ref131] Young K. Morrison H. (2018). Quantifying microglia morphology from photomicrographs of immunohistochemistry prepared tissue using ImageJ. J. Vis. Exp. 136:e57648. doi: 10.3791/57648, PMID: 29939190 PMC6103256

[ref132] Zajac D. Simpson J. Morganti J. M. Estus S. (2021). Expression of the microglial INPP5D isoforms as a function of Alzheimer’s disease status and genetics. Alzheimers Dement. 17:e056445. doi: 10.1002/alz.056445

[ref133] Zamani A. Powell K. L. May A. Semple B. D. (2020). Validation of reference genes for gene expression analysis following experimental traumatic brain injury in a pediatric mouse model. Brain Res. Bull. 156, 43–49. doi: 10.1016/j.brainresbull.2019.12.015, PMID: 31904409

[ref134] Zhang K. Bai X. Li R. Xiao Z. Chen J. Yang F.. (2012). Endogenous glucocorticoids promote the expansion of myeloid-derived suppressor cells in a murine model of trauma. Int. J. Mol. Med. 30, 277–282. doi: 10.3892/ijmm.2012.1014, PMID: 22664747

[ref135] Zhang Y. Chen K. Sloan S. A. Bennett M. L. Scholze A. R. O'keeffe S.. (2014). An Rna-sequencing transcriptome and splicing database of glia, neurons, and vascular cells of the cerebral cortex. J. Neurosci. 34, 11929–11947. doi: 10.1523/JNEUROSCI.1860-14.2014, PMID: 25186741 PMC4152602

[ref136] Zhang Y. Sloan S. A. Clarke L. E. Caneda C. Plaza C. A. Blumenthal P. D.. (2016). Purification and characterization of progenitor and mature human astrocytes reveals transcriptional and functional differences with mouse. Neuron 89, 37–53. doi: 10.1016/j.neuron.2015.11.013, PMID: 26687838 PMC4707064

[ref137] Zhao J. Bi W. Xiao S. Lan X. Cheng X. Zhang J.. (2019). Neuroinflammation induced by lipopolysaccharide causes cognitive impairment in mice. Sci. Rep. 9:5790. doi: 10.1038/s41598-019-42286-8, PMID: 30962497 PMC6453933

[ref138] Zhou H. Li X. M. Meinkoth J. Pittman R. N. (2000). Akt regulates cell survival and apoptosis at a postmitochondrial level. J. Cell Biol. 151, 483–494. doi: 10.1083/jcb.151.3.483, PMID: 11062251 PMC2185587

