# Supplementary material for: Regulation of microglial responses after pediatric traumatic brain injury: exploring the role of SHIP-1
Source: Front Neurosci. 2023 Oct 13;17:1276495. doi: 10.3389/fnins.2023.1276495 (PMC10603304; doi:10.3389/fnins.2023.1276495)
Supplement: Supplementary file 1 [file Data_Sheet_1.pdf]

Supplementary Figures

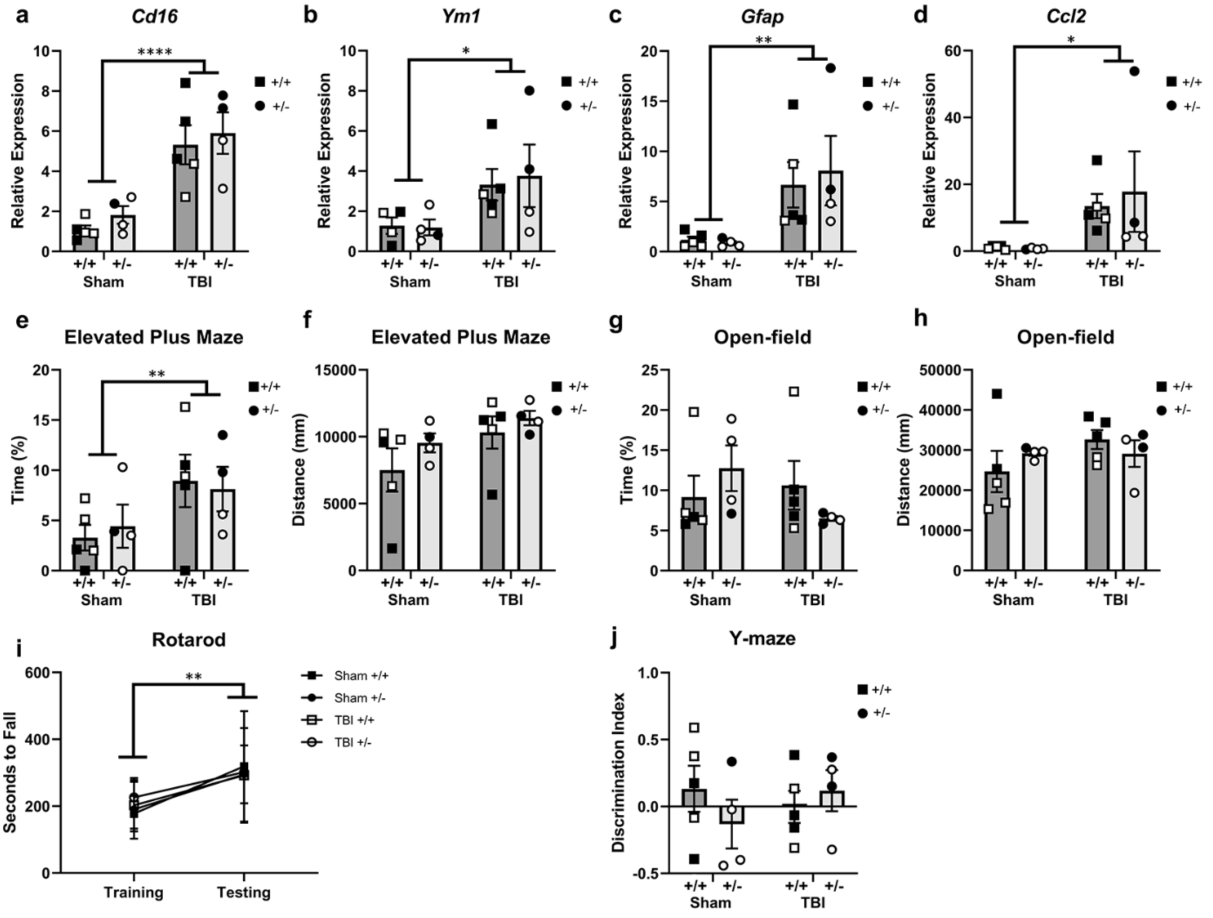

Supplementary Figure S1. SHIP-1+/+ and SHIP-1+/- had comparable immunological responses to brain injury and behavioral changes at 1-week post-injury. SHIP-1+/+ and SHIP-1+/- mice underwent experimental TBI surgeries at p21. Behavior and brains were collected at 1-week post-injury. (a-d) Relative expression of the indicated genes in the ipsilateral cortex compared to sham SHIP-1+/+ control animals. (e) Percentage time spent in the open arm and (f) total distance traveled during Elevated Plus Maze. (g) Percentage time spent in the center zone and (h) total distance traveled during open-field. (i) Seconds to fall during rotarod. (j) Discrimination index calculated from time spent in novel arm against familiar arm during y-maze. n=4-5/group. Solid = female, open = male. *$p < 0.05$, **$p < 0.005$, ****$p < 0.0001$. Two-way ANOVA and three-way ANOVA;

Supplementary Table S1. Statistical analysis of the expression of various genes in SHIP-1+/+ and SHIP-1+/- mice at 1-week post-injury, as well as percentage time spent in the open arm and total distance traveled during Elevated Plus Maze; time spent in the center zone and total distance traveled during open-field; seconds to fall during rotarod and discrimination index calculated from time spent in novel arm against familiar arm during y-maze (presented graphically in Supplementary Figure S1).

**Gene**	**Injury (F, p)**	**Genotype (F, p)**	**Time (F, p)**	**Interactions**
Fcgr3	$F_{1, 14} = 30.72$, $p < 0.0001$****	$F_{1, 14} = 0.78$, $p = 0.3913$	N/A	None
Chil3	$F_{1, 14} = 6.40$, $p = 0.0251$*	$F_{1, 14} = 0.04$, $p = 0.8484$	N/A	None
Gfap	$F_{1, 14} = 10.10$, $p = 0.0067$**	$F_{1, 14} = 0.07$, $p = 0.7895$	N/A	None
Ccl2	$F_{1,14} = 6.70$, $p = 0.0214$*	$F_{1,14} = 0.12$, $p = 0.7361$	N/A	None
**Measurement**	**Injury (F, p)**	**Genotype (F, p)**		**Interactions**
Time in open arm – Elevated Plus Maze	$F_{1, 14} = 4.81$, $p = 0.0456$*	$F_{1, 14} = 0.01$, $p = 0.9394$	N/A	None
Distance traveled – Elevated Plus Maze	$F_{1, 14} = 3.71$, $p = 0.0746$	$F_{1, 14} = 1.66$, $p = 0.2191$	N/A	None
Time in center zone – open-field test	$F_{1, 14} = 0.84$, $p = 0.3750$	$F_{1, 14} = 0.01$, $p = 0.9217$	N/A	None
Total distance traveled – open-field test	$F_{1,14} = 1.22$, $p = 0.2874$	$F_{1,14} = 0.022$, $p = 0.8844$	N/A	None
Seconds to fall – Rotarod test	$F_{1, 28} = 0.09$, $p = 0.7649$	$F_{1, 28} = 0.02$, $p = 0.8746$	$F_{1, 28} = 9.55$, $p = 0.0045$**	None
Discrimination index – Y-maze test	$F_{1, 14} = 0.12$, $p = 0.2028$	$F_{1, 14} = 0.20$, $p = 0.6594$	N/A	None

Supplementary Table S2. The number of pediatric SHIP-1+/- and SHIP-1-/- mice allocated to each procedure and their respective sex distribution within each cohort (F = female; M = male).

**Pediatric TBI Animal Numbers**			
**Acute**			
**Procedure**	**Injury Type**	**Genotype**	
		Control (SHIP-1+/-)	Knockout (SHIP-1-/-)
Behavior	Sham	n=15 (F=6; M=9)	n=11 (F=2; M=9)
	Severe TBI	n=17 (F=5; M=12)	n=12 (F=6; M=6)
Histology	Sham	n=6 (F=5; M=1)	n=5 (F=2; M=3)
	Severe TBI	n=7 (F=5; M=2)	n= 6 (F=2; M=4)
Gene Expression (6 Hours Post-injury)	Sham	n=5 (F=2; M=3)	n=5 (F=4; M=1)
	Severe TBI	n=6 (F=4; M=2)	n=6 (F=2; M=4)
Gene Expression (1 Week Post-injury)	Sham	n=5 (F=2; M=3)	n=6 (F=2; M=4)
	Severe TBI	n=6 (F=2; M=4)	n=5 (F=3; M=2)
**Chronic**			
**Procedure**	**Injury Type**	**Genotype**	
		Control (SHIP-1+/-)	Knockout (SHIP-1-/-)
Behavior	Sham	n=15 (F=6; M=9)	n=9 (F=1; M=8)
	Severe TBI	n=17 (F=5; M=12)	n=10 (F=6; M=4)
Histology	Sham	n=6 (F=3; M=3)	n=5 (F=1; M=4)
	Severe TBI	n=6 (F=3; M=3)	n= 6 (F=3; M=3)
Gene Expression	Sham	n=5 (F=2; M=3)	n=4 (F=2; M=2)
	Severe TBI	n=4 (F=1; M=3)	n= 5 (F=3; M=2)
Serum Cytokines	Sham	n=6 (F=3; M=3)	n=6 (F=1; M=5)
	Severe TBI	n=6 (F=3; M=3)	n= 6 (F=3; M=3)

Supplementary Table S3. Gene names and assay ID used for quantitative polymerase chain reaction experiments.

**Marker**	**Gene Name**	**Gene Assay ID (Thermo Fisher)**
Chemokine (C-C Motif) Ligand 2 (CCL2)	*Ccl2*	Mm00441242_m1
CD16	*Fcgr3*	Mm00438882_m1
CD68	*Cd68*	Mm03047343_m1
CD86	*Cd86*	Mm00444540_m1
CD206	*Mrc1*	Mm01329362_m1
Corticotrophin-releasing Hormone	*Crh*	Mm01293920_s1
C-X3-C Motif Chemokine Receptor 1 (CX3CR1)	*Cx3cr1*	Mm00438354_m1
Excitatory Amino Acid Transporter 2 (SLC1A2)	*Slc1a2*	Mm01275814_m1
Interleukin 1 beta (IL-1β)	*Il1b*	Mm00434228_m1
Ionized Calcium-binding Adapter Molecule 1 (IBA-1)	*Aif1*	Mm00479862_g1
Insulin Growth Factor Binding Protein 7 (IGFBP7)	*Igfbp7*	Mm03807886_m1
Glial Fibrillary Acidic Protein (GFAP)	*Gfap*	Mm01253033_m1
Multiple EGF Like Domains 10	*Megf10*	Mm01257625_m1
MHC Class II	*H2-ab1*	Mm00439216_m1
Matrix Metallopeptidase 12	*Mmp12*	Mm00500554_m1
Reduced Nicotinamide Adenine Dinucleotide Phosphate (NADPH)	*Nox2*	Mm01287743_m1
Spalt Like Transcription Factor 1 (SALL1)	*Sall1*	Mm00491266_m1
Src Homology 2 Domain Containing Inositol Polyphosphate 5-phosphate 1 (SHIP-1)	*Inpp5d*	Mm00494963_m1
Transforming Growth Factor β (TGF-β)	*Tgfb*	Mm01178820_m1
Translocator Protein (TSPO)	*Tspo*	Mm00437828_m1
TMEM119	*Tmem119*	Mm00525305_m1
TREM2	*Trem2*	Mm04209424_g1

Ym1	*Chil3*	Mm00657889_mH

.

Supplementary Table S4. Statistical analysis of the expression of various genes in SHIP-1+/- and SHIP-1-/- mice at 6 hours post-injury (presented graphically in Figure 1).

Gene	Injury (F, p)	Genotype (F, p)	Interactions
Tnf	$F_{1, 15}$ = 7.37, p = 0.0160*	$F_{1, 15}$ = 0.76, p = 0.3973	None
Ccl-2	$F_{1, 18}$ = 8.98, p = 0.0077**	$F_{1, 18}$ = 0.46, p = 0.5065	None
Il1b	$F_{1, 16}$ = 3.54, p = 0.0780*	$F_{1, 16}$ = 1.26, p = 0.2781	None
Chil3	$F_{1,18}$ = 9.64, p = 0.0061**	$F_{1,18}$ = 8.05, p = 0.0109*	None
H2-ab1	$F_{1,18}$ = 5.83, p = 0.0266*	$F_{1,18}$ = 0.01, p = 0.9064	None
Gfap	$F_{1, 17}$ = 6.15, p = 0.0239*	$F_{1, 17}$ = 0.59, p = 0.4519	None

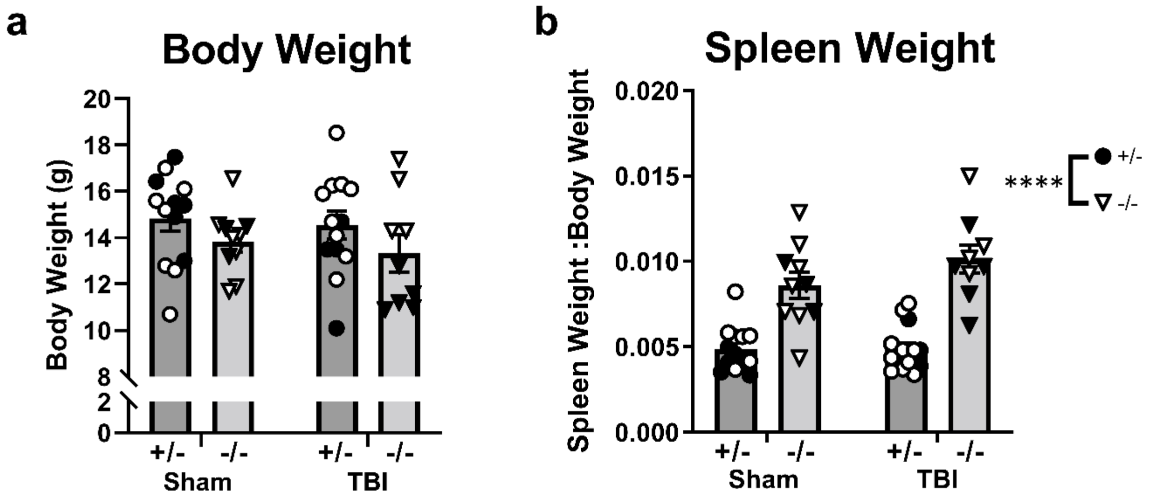

Supplementary Figure S2. Changes in body weight and spleen weight at 1-week post-injury. (a) Body weight and (b) spleen weight of SHIP-1+/- and SHIP-1-/- mice at 1-week post-injury. ****$p < 0.0001$. Two-way ANOVA. n=10-17/group. Solid = female, open = male. Complete statistical analysis is presented in supplementary table S4.

Supplementary Table S5. Statistical analysis of body weight, spleen weight relative to body weight, IBA-1 fluorescence, number of IBA-1+ microglia and microglial morphology analysis at 1-week post-injury (presented graphically in figure 2).

Measurements	Injury (F, p)	Genotype (F, p)	Interactions
Body weight	$F_{1,41} = 0.40$, $p = 0.5293$	$F_{1,41} = 3.24$, $p = 0.0793$	None
Spleen weight	$F_{1,42} = 1.92$, $p = 0.1731$	$F_{1,42} = 65.0$, $p < 0.0001$****	None
IBA-1 fluorescence – ipsilateral cortex	$F_{1,20} = 32.58$, $p < 0.0001$****	$F_{1,20} = 0.02$, $p = 0.9031$	None
IBA-1 fluorescence – ipsilateral peri-lesional cortex	$F_{1, 20} = 40.48$, $p < 0.0001$****	$F_{1,20} = 0.46$, $p = 0.5057$	None
IBA-1 fluorescence – ipsilateral dentate gyrus	$F_{1,20} = 20.29$, $p = 0.0002$***	$F_{1,20} = 0.29$, $p = 0.5972$	None
IBA-1 fluorescence – ipsilateral dorsolateral thalamus	$F_{1,20} = 21.57$, $p = 0.0002$***	$F_{1,20} = 0.19$, $p = 0.5650$	None
Number of IBA-1+ microglia - ipsilateral cortex	$F_{1,20} = 54.87$, $p < 0.0001$****	$F_{1,20} = 0.36$, $p = 0.5570$	None
Number of IBA-1+ microglia – ipsilateral peri-lesional cortex	$F_{1,20} = 66.79$, $p < 0.0001$****	$F_{1,20} = 0.21$, $p = 0.6533$	None
Number of IBA-1+ microglia – ipsilateral dentate gyrus	$F_{1,20} = 43.60$, $p < 0.0001$****	$F_{1,20} = 0.54$, $p = 0.4727$	None
Number of IBA-1+ microglia – ipsilateral dorsolateral thalamus	$F_{1,20} = 54.81$, $p < 0.0001$****	$F_{1,20} = 0.22$, $p = 0.6433$	None
Microglial morphology - average branch length	$F_{1,21} = 11.07$, $p = 0.0032$**	$F_{1,21} = 0.02$, $p = 0.9019$	None
Microglial morphology - number of branches	$F_{1,21} = 9.14$, $p = 0.0065$**	$F_{1,21} = 2.23$, $p = 0.1498$	None
Microglial morphology - soma area	$F_{1,21} = 15.43$, $p = 0.0008$***	$F_{1,21} = 0.92$, $p = 0.3492$	None

Supplementary Table S6. Statistical analysis of the expression of various genes in SHIP-1+/- and SHIP-1-/- mice at 1-week post-injury (presented graphically in figure 3).

Gene	Injury (F, p)	Genotype (F, p)	Interactions
Cd86	$F_{1,17}$ = 1.47, p = 0.2421	$F_{1,17}$ = 0.34, p = 0.5685	None
Fcgr3	$F_{1,16}$ = 3.79, p = 0.0694	$F_{1,16}$ = 7.83, p = 0.0129*	None
Mrc1	$F_{1,15}$ = 1.08, p = 0.05769	$F_{1,15}$ = 7.93, p = 0.0130*	None
Tmem119	$F_{1,19}$ = 0.75, p = 0.3969	$F_{1,19}$ = 2.30, p = 0.1455	None
Trem2	$F_{1,20}$ = 1.96, p = 0.1765	$F_{1,20}$ = 1.52, p = 0.2312	None
Sall1	$F_{1,20}$ = 0.41, p = 0.5278	$F_{1,20}$ = 5.26, p = 0.03280*	None

Supplementary Table S7. Statistical analysis of CD68 and GFAP fluorescence, and *Ccl2* gene expression at 1-week post-injury (presented graphically in figure 4 and supplementary figure S3).

**Measurement**	**Injury (F, p)**	**Genotype (F, p)**	**Interactions**
CD68 fluorescence – injury site	$F_{1,20} = 11.84$, $p = 0.0026$**	$F_{1,20} < 0.00$, $p = 0.9681$	None
CD68 fluorescence – ipsilateral corpus callosum/external capsule	$F_{1,20} = 30.93$, $p < 0.0001$****	$F_{1,20} = 0.020$, $p = 0.8900$	None
GFAP fluorescence – injury site	$F_{1,21} = 54.90$, $p < 0.0001$****	$F_{1,21} = 1.51$, $p = 0.2328$	None
Ccl2 gene expression	$F_{1,18} = 4.97$, $p = 0.0388$	$F_{1,18} = 0.31$, $p = 0.5844$	None

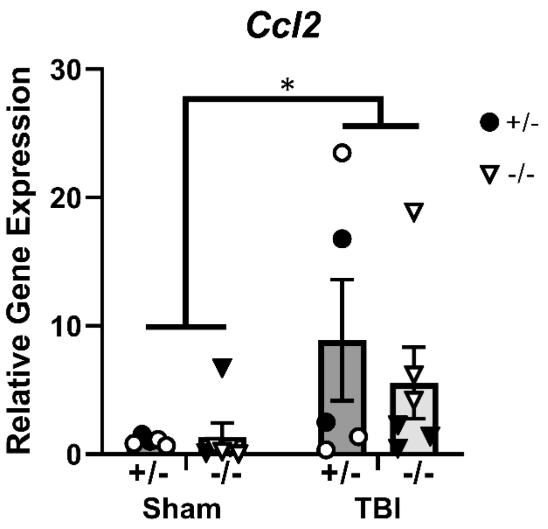

Supplementary Figure S3. *Ccl2* gene expression relative to sham SHIP-1+/- control mice at the cortex at of SHIP-1+/- and SHIP-1-/- mice at 1-week post-injury. *p < 0.05. Two-way ANOVA; n=4-6/group. Solid = female, open = male. Complete statistical analysis is presented in supplementary table S6.

Supplementary Table S8. Statistical analysis of remaining healthy tissue at the cortex and hippocampus, measurements of anxiety-like behavior, explorative tendency, motor skills and working memory at 1-week post-injury (presented graphically in figure 5).

Measurement	Injury (F, p)	Genotype (F, p)	Time (F,p)	Interactions
Healthy remaining tissue - cortex	$F_{1,21} = 50.79$, $p < 0.0001$****	$F_{1,21} = 2.10$, $p < 0.1619$	N/A	None
Healthy reaming tissue – hippocampus	$F_{1,21} = 1.73$, $p = 0.2025$	$F_{1,21} = 1.32$, $p = 0.2628$	N/A	None
Time in open arm – Elevated Plus Maze	$F_{1,54} = 0.10$, $p = 0.8822$	$F_{1,54} = 1.87$, $p = 0.9127$	N/A	None
Distance travelled – Elevated Plus Maze	$F_{1,54} = 1.30$, $p = 0.2585$	$F_{1,54} = 5.97$, $p = 0.0178$*	N/A	None
Time in center zone – open-field test	$F_{1,55} = 13.44$, $p = 0.0006$***	$F_{1,55} = 4.49$, $p = 0.0387$*	N/A	Injury x genotype $F_{1,55} = 5.68$, $p = 0.0206$*
Total distance traveled – open-field test	$F_{1,55} = 0.05$, $p = 0.8269$	$F_{1,55} = 8.11$, $p = 0.0062$**	N/A	Injury x genotype $F_{1,55} = 5.52$, $p = 0.0224$*
Seconds to fall – Rotarod test	$F_{1,54} = 1.04$, $p = 0.3134$	$F_{1,54} = 1.25$, $p = 0.2680$	$F_{1,54} = 19.39$, $p < 0.0001$****	None
Discrimination index – Y-maze test	$F_{1,54} = 4.02$, $p = 0.0501$	$F_{1,54} = 0.05$, $p = 0.8309$	N/A	None

Supplementary Table S9. Statistical analysis of body weight, spleen weight and serum cytokine concentration of SHIP-1+/- and SHIP-1-/- mice at 12-weeks post-injury (presented graphically in figure 6).

**Measurement**	**Injury (F, p)**	**Genotype (F, p)**	**Interactions**
Body weight	$F_{1,49}$ = 2.20, p = 0.1443	$F_{1,49}$ = 19.40, p < 0.0001****	None
Spleen weight	$F_{1, 49}$ = 1.04, p = 0.3131	$F_{1, 49}$ = 65.38, p < 0.0001****	None
**Serum Cytokine**	**Injury (F, p)**	**Genotype (F, p)**	**Interactions**
G-CSF	$F_{1,20}$ = 0.04, p = 0.8379	$F_{1,20}$ = 5.22, p = 0.0334*	None
IL-4	$F_{1,20}$ = 0.57, p = 0.4592	$F_{1,20}$ = 15.83, p = 0.0007***	None
IL-6	$F_{1,20}$ = 0.28, p = 0.6021	$F_{1,20}$ = 7.02, p = 0.0154*	None
IFN-γ	$F_{1,20}$ = 7.39, p = 0.0132*	$F_{1,20}$ = 2.22, p = 0.1520	None
IL-12(p40)	$F_{1,20}$ < 0.00, p = 0.9889	$F_{1,20}$ = 25.24, p < 0.0001****	None
IL-10	$F_{1,20}$ = 7.20, p = 0.0142*	($F_{1,20}$ = 50.83, p < 0.0001****	None
MIP-1α	$F_{1,20}$ = 6.13, p = 0.0224*	$F_{1,20}$ = 40.40, p < 0.0001****	None
MIP-1β	$F_{1,20}$ = 3.141, p = 0.0916	$F_{1,20}$ = 8.81, p = 0.0076*	None

Supplementary Table S10. Statistical analysis of gene expression analysis of the cortex and hippocampus of SHIP-1+/- and SHIP-1-/- mice at 12-weeks post-injury (presented graphically in figure 7).

**Cortex**			
**Gene**	**Injury (F, p)**	**Genotype (F, p)**	**Interactions**
Inpp5d	$F_{1,16} = 0.74$,<br>$p = 0.4022$	$F_{1,16} = 71.37$,<br>$p < 0.0001$****	None
Cd86	$F_{1,16} = 0.61$,<br>$p = 0.4492$	$F_{1,16} = 25.12$,<br>$p < 0.0001$****	$F_{1,16} = 7.24$,<br>$p = 0.0161$*
Nadph	$F_{1,16} = 0.13$,<br>$p = 0.7178$	$F_{1,16} = 15.24$,<br>$p = 0.0013$**	None
Mcr1	$F_{1,16} < 0.00$,<br>$p = 0.9795$	$F_{1,16} = 3.940$,<br>$p = 0.0646$	Injury x genotype<br>$F_{1,16} = 4.76$,<br>$p = 0.0444$*
Megf10	$F_{1,16} = 0.0345$,<br>$p = 0.8550$	$F_{1,16} = 5.918$,<br>$p = 0.0271$*	None
**Hippocampus**			
**Gene**	**Injury (F, p)**	**Genotype (F, p)**	**Interactions**
Cd86	$F_{1,16} = 0.08$,<br>$p = 0.7817$	$F_{1,16} = 19.59$,<br>$p = 0.0004$***	None
Nadph	$F_{1,16} = 12.73$,<br>$p = 0.0026$***	$F_{1,16} = 15.72$,<br>$p = 0.0011$***	None
Mcr1	$F_{1,16} = 8.003$,<br>$p = 0.0121$*	$F_{1,16} < 0.00$,<br>$p = 0.9731$	None
Megf10	$F_{1,16} = 3.73$,<br>$p = 0.0714$	$F_{1,16} = 4.21$,<br>$p = 0.0568$	None
Cd68	$F_{1,16} = 28.04$,<br>$p < 0.0001$****	$F_{1,16} = 12.45$,<br>$p = 0.0028$***	Injury x genotype<br>$F_{1,16} = 7.28$,<br>$p = 0.0158$*
Cx3cr1	$F_{1,16} = 4.67$,<br>$p = 0.0462$*	$F_{1,16} = 11.85$,<br>$p = 0.0033$**	None
Ccl2	$F_{1,16} = 4.64$,<br>$p = 0.0468$*	$F_{1,16} = 21.67$,<br>$p = 0.0003$***	None
Mmp12	$F_{1,16} = 6.94$,<br>$p = 0.0187$*	$F_{1,16} = 1.54$,<br>$p = 0.2334$	None
Crh	$F_{1,16} = 21.19$,<br>$p = 0.0003$***	$F_{1,16} = 1.18$,<br>$p = 0.2930$	None
Tspo	$F_{1,16} = 7.64$,<br>$p = 0.0138$*	$F_{1,16} = 6.52$,<br>$p = 0.0213$*	None

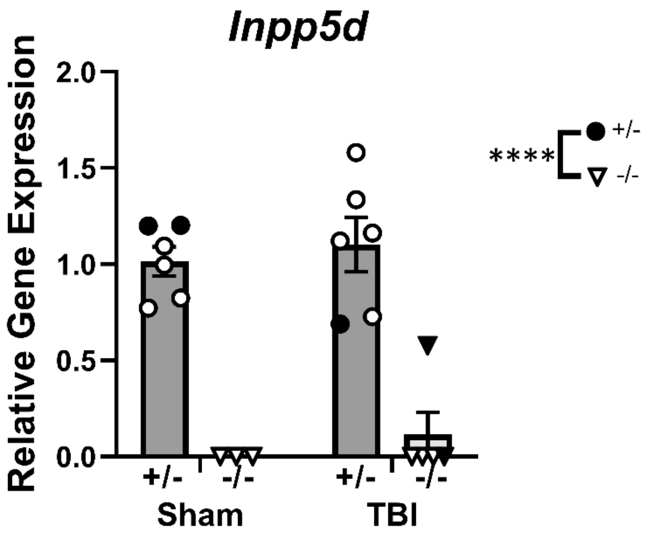

Supplementary Figure S4. *Inpp5d* expression relative to sham SHIP-1+/- mice in the ipsilateral cortex of SHIP-1+/- and SHIP-1-/- mice at 12-weeks post-injury. ****p < 0.0001. Two-way ANOVA; n=3-6/group. Solid = female, open = male. Statistics presented in Supplementary Table S9.

Supplementary Table S11. Statistical analysis of IBA-1 fluorescence, number of IBA-1+ microglia, microglial morphology and GFAP fluorescence of SHIP-1+/- and SHIP-1-/- mice at 12-weeks post-injury (presented graphically in figures 8, 9 and 10).

Measurements	Injury (F, p)	Genotype (F, p)	Interactions
IBA-1 fluorescence – ipsilateral cortex	$F_{1,19} = 0.08$, $p = 0.7812$	$F_{1,19} = 1.66$, $p = 0.2134$	None
IBA-1 fluorescence – ipsilateral peri-lesional cortex	$F_{1,19} < 0.00$, $p = 0.9730$	$F_{1,19} = 1.83$, $p = 0.1925$	None
IBA-1 fluorescence – ipsilateral dentate gyrus	$F_{1,18} = 0.17$, $p = 0.6864$	$F_{1,18} < 0.00$, $p = 0.9390$	None
IBA-1 fluorescence – ipsilateral dorsolateral thalamus	$F_{1,19} = 0.04$, $p = 0.8494$	$F_{1,19} = 0.36$, $p = 0.5567$	None
Number of iba-1+ microglia - ipsilateral cortex	$F_{1,19} = 0.49$, $p = 0.4943$	$F_{1,19} = 1.31$, $p = 0.7216$	None
Number of IBA-1+ microglia – ipsilateral peri-lesional cortex	$F_{1,19} = 0.37$, $p = 0.5526$	$F_{1,19} = 1.23$, $p = 0.2810$	None
Number of IBA-1+ microglia – ipsilateral dentate gyrus	$F_{1,18} = 1.20$, $p = 0.2881$	$F_{1,19} = 0.07$, $p = 0.4118$	None
Number of IBA-1+ microglia – ipsilateral dorsolateral thalamus	$F_{1,18} = 1.49$, $p = 0.2379$	$F_{1,19} = 1.08$, $p = 0.3125$	None
Microglial morphology – number of branches	$F_{1,15} = 3.22$, $p = 0.5791$	$F_{1,15} = 1.82$, $p = 0.1971$	None
Microglial morphology – number of endpoints	$F_{1,15} = 0.49$, $p = 0.4961$	$F_{1,15} = 2.17$, $p = 0.1664$	None
Microglial morphology – branch length	$F_{1,15} < 0.00$, $p = 0.9841$	$F_{1,15} = 3.61$, $p = 0.0768$	None
GFAP fluorescence – ipsilateral cortex	$F_{1,19} = 46.16$, $p < 0.0001$****	$F_{1,19} = 6.33$, $p = 0.0210$*	Injury x genotype $F_{1,19} = 6.16$, $p = 0.0225$*

Supplementary Table S12. Statistical analysis of remaining healthy tissue at the cortex and hippocampus, measurements of anxiety-like behavior, explorative tendency, motor skills and working memory at 12-week post-injury (presented graphically in figure 11).

Measurement	Injury (F, p)	Genotype (F, p)	Time (F,p)	Interactions
Healthy remaining tissue - cortex	$F_{1,19} = 441.10$, $p < 0.001$****	$F_{1,19} = 3.12$, $p = 0.0935$	N/A	None
Healthy reaming tissue – hippocampus	$F_{1,19} = 31.65$, $p < 0.001$****	$F_{1,19} = 17.0$, $p = 0.6827$	N/A	None
Time in open arm – Elevated Plus Maze	$F_{1,49} = 0.018$, $p = 0.8937$	$F_{1,49} = 2.54$, $p = 0.1176$	N/A	None
Distance traveled – Elevated Plus Maze	$F_{1,49} = 0.30$, $p = 0.5858$	$F_{1,49} = 15.88$, $p = 0.0002$***	N/A	None
Time in center zone – open-field test	$F_{1,49} = 0.18$, $p = 0.6768$	$F_{1,49} = 0.72$, $p = 0.3999$	N/A	Injury x genotype $F_{1,55} = 5.68$, $p = 0.0206$*
Total distance traveled – open-field test	$F_{1,49} = 1.488$, $p = 0.2284$	$F_{1,49} = 10.76$, $p = 0.0019$**	N/A	Injury x genotype $F_{1,55} = 5.52$, $p = 0.0224$*
Seconds to fall – Rotarod test	$F_{1,43} = 0.89$, $p = 0.3498$	$F_{1,43} = 1.20$, $p = 0.6637$	$F_{1,86} = 1.21$, $p = 0.2735$	None
Discrimination index – Y-maze test	$F_{1,49} = 0.57$, $p = 0.4525$	$F_{1,49} = 4.21$, $p = 0.0457$*	N/A	None
